# Caveolin 1 is required for axonal outgrowth of motor neurons and affects *Xenopus* neuromuscular development

**DOI:** 10.1038/s41598-020-73429-x

**Published:** 2020-10-05

**Authors:** Marlen Breuer, Hanna Berger, Annette Borchers

**Affiliations:** 1 Department of Biology, Molecular Embryology, Philipps-University Marburg, Marburg, Germany; 2 DFG Research Training Group, Membrane Plasticity in Tissue Development and Remodeling, GRK 2213, Philipps-University Marburg, Marburg, Germany

**Keywords:** Cell biology, Developmental biology

## Abstract

Caveolins are essential structural proteins driving the formation of caveolae, specialized invaginations of the plasma membrane. Loss of Caveolin-1 (Cav1) function in mice causes distinct neurological phenotypes leading to impaired motor control, however, the underlying developmental mechanisms are largely unknown. In this study we find that loss-of-function of *Xenopus* Cav1 results in a striking swimming defect characterized by paralysis of the morphants. High-resolution imaging of muscle cells revealed aberrant sarcomeric structures with disorganized actin fibers. As *cav1* is expressed in motor neurons, but not in muscle cells, the muscular abnormalities are likely a consequence of neuronal defects. Indeed, targeting *cav1* Morpholino oligonucleotides to neural tissue, but not muscle tissue, disrupts axonal outgrowth of motor neurons and causes swimming defects. Furthermore, inhibition of voltage-gated sodium channels mimicked the Cav1 loss-of-function phenotype. In addition, analyzing axonal morphology we detect that Cav1 loss-of-function causes excessive filopodia and lamellipodia formation. Using rescue experiments, we show that the Cav1 Y14 phosphorylation site is essential and identify a role of RhoA, Rac1, and Cdc42 signaling in this process. Taken together, these results suggest a previously unrecognized function of Cav1 in muscle development by supporting axonal outgrowth of motor neurons.

## Introduction

Caveolin proteins are versatile integral membrane proteins involved in a broad spectrum of cellular processes. They form the main structural component of caveolae, which are flask-shaped plasma membrane invaginations of membrane lipid raft domains^1–4^. As components of the endocytic machinery, caveolae as well as their Caveolin proteins are involved in a broad spectrum of cellular processes, by regulating the activity, compartmentalization and internalization of signaling molecules in the context of cell proliferation, survival as well as cellular integrity^5,6^. Furthermore, they also play a role during mechanoprotection by acting as stretch-sensors and serving as membrane reservoirs during mechanical stress^7–9^.

The Caveolin protein family consists of three isoforms—Cav1 Cav2 and Cav3—which share high similarities in their membrane topology and are able to oligomerize to heteromeric complexes forming the caveolae coat^10–14^. Caveolin proteins are widely expressed in many cell types with high abundance in fibroblasts, adipocytes, endothelial and muscle cells, whereas Cav3 is muscle-specific^3,11,15–17^. Cav1 is expressed as α- and β-isoform. The Cav1α isoform is considered to represent the full-length protein, whereas the β-isoform lacks the first 32 N-terminal amino acids of the α-isoform^18,19^. In humans, mice and zebrafish the Cav1α and β isoforms are encoded by the same gene but are translated from distinct mRNAs generated by alternative splicing^18–20^. In *Xenopus*, the Cav1 isoforms are encoded on separate chromosomes, chromosome 3L and the homologous short chromosome 3S. The full-length Cav1α isoform is encoded on chromosome 3L and here referred to as Cav1L.

The role of Caveolin proteins in neurons has sparked interest, as the existence of caveolae and classical caveolin-mediated endocytosis has been controversially debated^21–25^. Furthermore, miss-expression of Cav1 has been linked to the progression of a variety of neurodegenerative diseases including Alzheimer, Schizophrenia, Huntington and also brain tumors^26–30^. Consistently, loss of function of Cav1 in mice results in neurodegenerative phenotypes such as accelerated neuronal aging, deficits in motor coordination, gait abnormalities and muscle weakness^29,31,32^. In addition, Cav1 has been shown to regulate early steps of neurogenesis by mediating caveolae-independent trafficking of L1 and N-cadherin during early neuronal migration and maturation in the cerebral cortex^33^. Moreover, Cav1 may also play a role in later aspects of neuronal development. For example, neuron-targeted Cav1 has been shown to improve synaptic plasticity in cultured hippocampal neurons, preserve memory and restore motor function induced by brain trauma in the ALS (Amyotrophic Lateral Sclerosis) mouse model^31,34–36^. However, so far, the role of Cav1 during distinct aspects of neurodevelopment is not fully understood.

## Results

### Cav1L loss-of-function affects the swimming behavior of *Xenopus* tadpoles

**Figure 1:**
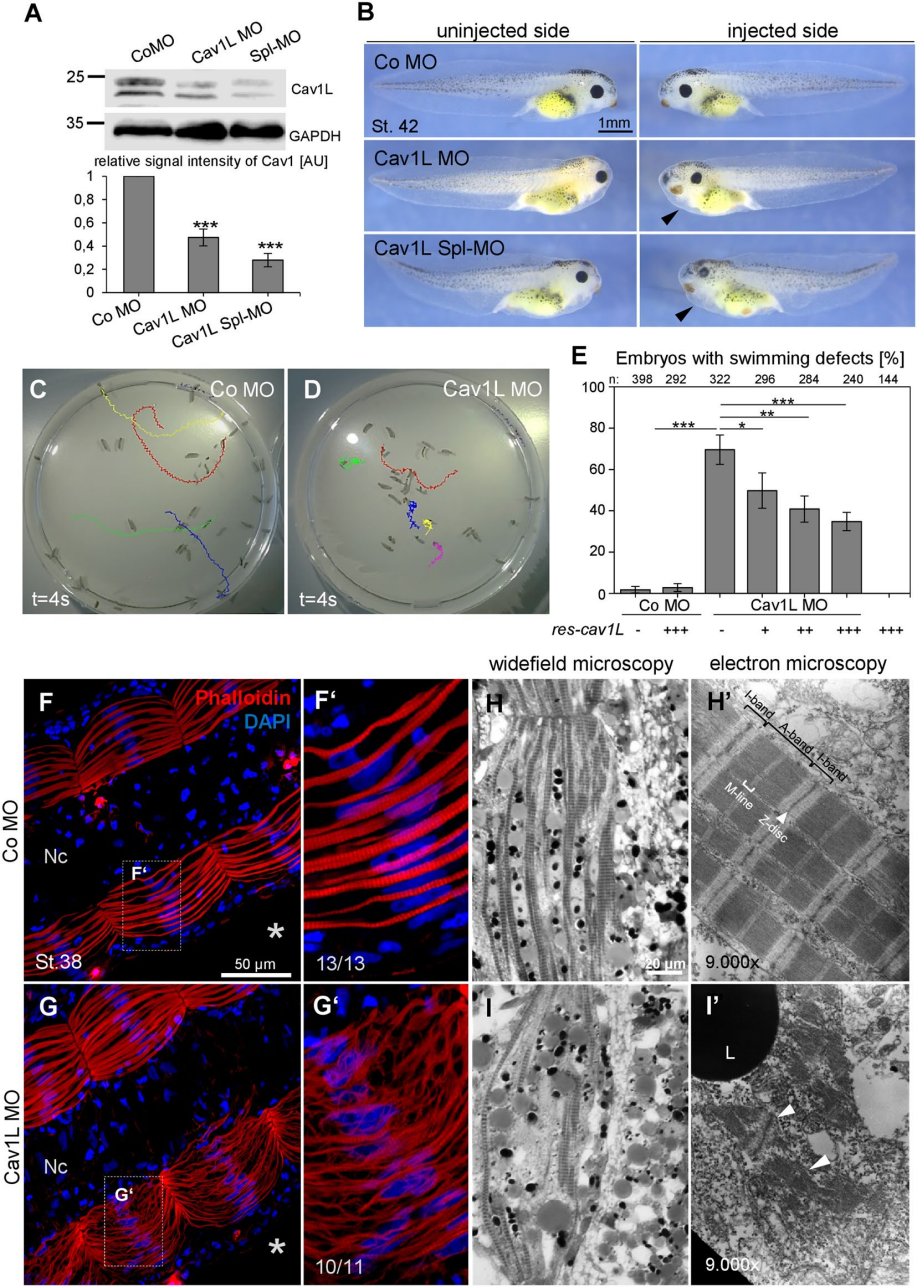
Loss-of-function of Cav1L affects sarcomeric organization and causes swimming defects. (**A**) Cav1L MO and Cav1L Spl-MO inhibit *Xenopus* Cav1L expression. *Xenopus* embryos were injected with 20 ng Morpholinos at the one-cell stage and Cav1α and GAPDH expression was analyzed by Western blotting at stage 27. The graph shows the relative Cav1α expression of four independent experiments, in relation to GAPDH expression and normalized to Co MO-injected embryos, data are mean ± s.e.m. ***p-value ≤ 0.001 (Student’s t-test). (**B**) *Xenopus* embryos were injected with 20 ng Morpholinos and *mGFP* RNA in one blastomere at the two-cell stage and analyzed at stage 42. Cav1L morphants show mild morphological abnormalities including a shortening of the anterior–posterior axis, craniofacial malformations and edema formation (arrowhead). (**C**,**D**) Tracks of the swimming movement (time frame 4 s) of controls (**C**) and morphant embryos (**D**). (**E**) Percentage of swimming defects of embryos injected unilaterally with Morpholinos (5–7.5 ng) alone or in combination with *res-cav1L* RNA (+ = 100 pg; +  +  = 200 pg, +  +  +  = 300 pg), a Cav1L-construct lacking the Morpholino binding side, at the two-cell stage. Number of analyzed embryos is indicated for each column. Data from at least three experiments are presented as the mean ± s.e.m. * p-value ≤ 0.05; **p-value ≤ 0.01; ***p-value ≤ 0.001 (Student’s t-test). (**F**,**G**) Muscle morphology of stage 38 embryos, injected unilaterally with 20 ng MO at the two-cell stage; asterisks mark injected side. Phalloidin staining reveals sarcomeric actin; DAPI staining marks the nuclei. (**F’**,**G’**) Higher magnification of the boxed areas in (**F**,**G**). Controls show normal sarcomeric actin organization, while morphants display wavy and disorganized actin fibers. (**H,I**) Single somitic segment of a control embryo (**H**) or a Cav1L morphant (**I**), injected with 10 ng MO in both blastomeres at the two-cell stage, showing disrupted actin organization within muscle cells. (**H’,I’**) Transmission electron microscopy (TEM) picture of control muscle cells with normal sarcomeric organization (**H’**) and Cav1L-morphant muscle cells, which do not show the characteristic sarcomeric structure. Arrowhead marks detached actin bundles; *L* lipid droplet, *Nc* notochord.

Here we used the *Xenopus* system to further dissect the in vivo function of Cav1 in neuromuscular development. First loss-of-function experiments were performed. *Xenopus laevis* embryos were injected in one blastomere at the two-cell stage with either a translation-blocking Morpholino (Cav1L MO) or a splice-blocking Morpholino (Cav1L Spl-MO) causing a deletion of the second exon (Supplementary Fig. S1A,B). Both Morpholinos significantly inhibited the expression of Cav1L compared to embryos injected with a control Morpholino (Co MO) as analyzed by Western blotting (Fig. 1A). The embryos displayed a minor shortening of the anterior–posterior axis (Fig. 1B), which is likely caused by convergent extension defects as recently shown by Putzig et al.^37^. However, as we did not target the injections to the mesoderm, we only observed mild morphological abnormalities. Nevertheless, the embryos showed severe swimming defects. While control embryos responded to a tactile stimulus by swimming straightforward, unilaterally injected Cav1L-morphant embryos moved in circles, caused by the paralysis of the injected side (Fig. 1C,D, supplementary Movie 1, 2, 3) or were completely paralyzed when injected into both blastomeres (supplementary Movie 4, 5). Injection of the translation-blocking Morpholino as well as the splice-blocking Morpholino caused a comparable percentage of defects, which were rescued by overexpression of a Cav1L construct lacking the Morpholino binding sites (res-cav1L) or wild-type cav1L, respectively (Fig. 1E, supplementary Fig. S1C). Thus, Cav1L seems to be required for embryo motility.

### Cav1L loss-of-function results in muscular defects

Embryonic motility can be affected by defects in muscular development as well as neuromuscular development. To analyze if the musculature of Cav1L-morphant tadpoles is affected, muscular actin was visualized using Phalloidin (Fig. 1F,G). Embryos injected with the Co MO showed a highly organized and characteristic striped pattern (Fig. 1F,F’). However, loss of Cav1L function led to a disorganized actin cytoskeleton and defects in muscular integrity (Fig. 1G,G’). These data were confirmed by ultra-structure analysis of the muscle fibers revealing the characteristic striped pattern of the sarcomeric bundles in controls (Fig. 1H,H’), while the sarcomeric ultrastructure was highly disrupted in Cav1L-morphant embryos displaying disorganized actin and myosin filaments (Fig. 1I,I’). Interestingly, somitogenesis per se seems not to be affected, as expression of *myoD* was not compromised in Cav1L morphants (supplementary Fig. S2). Taken together, knockdown of Cav1L results in severe muscular defects, which are likely the cause of the aberrant swimming behavior.

### Cav1L is expressed in neural tissue and loss-of-function indicates a function in neural but not muscle tissue

**Figure 2:**
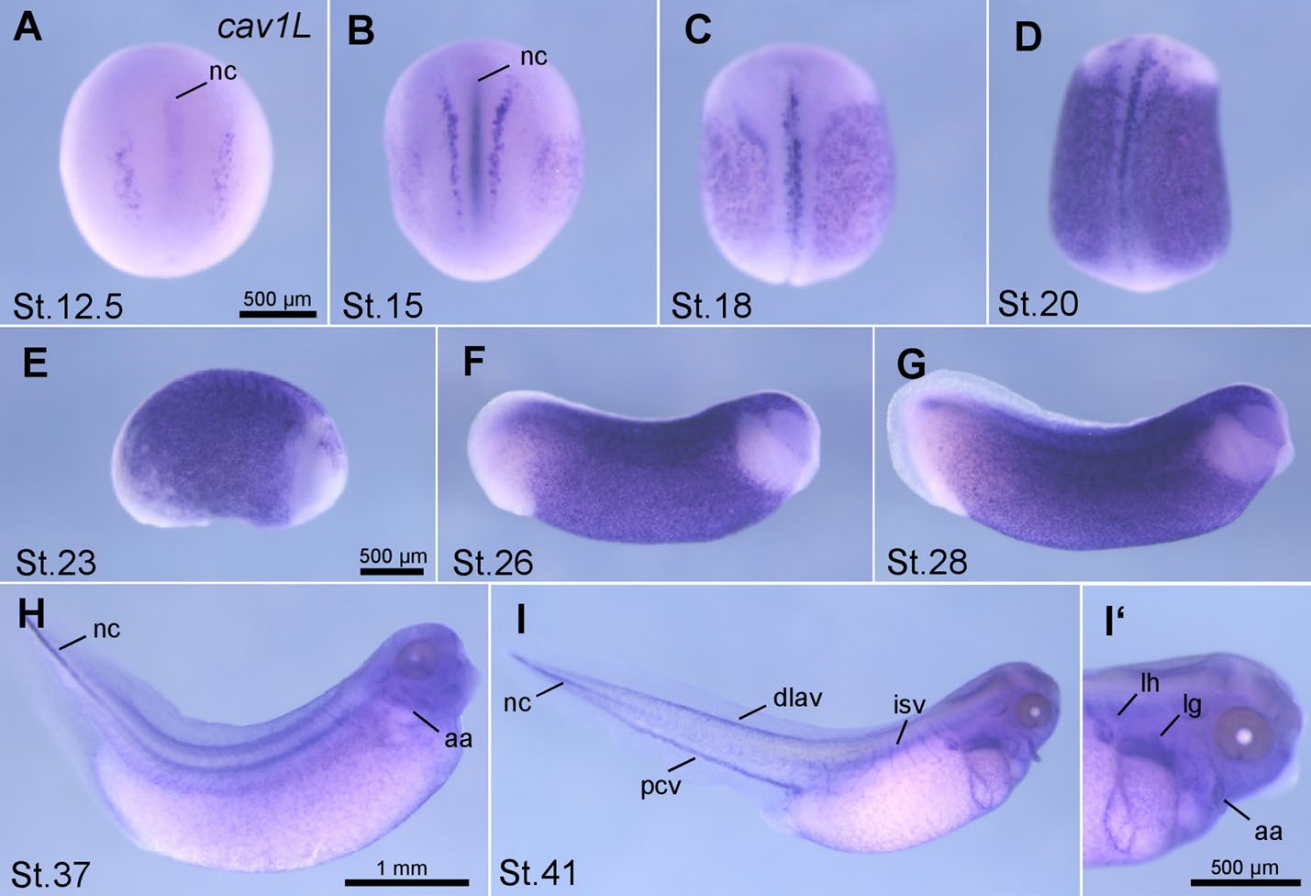
*Cav1L* is expressed in the notochord and the cardio-vasculature during *Xenopus* development Temporal and spatial *cav1L* expression analyzed by in situ hybridization. (**A**) Dorsal view of a stage 12.5 embryo, *cav1L* is detected in the notochord and two thin stripes on both sides at the dorsal midline. (**B**) Dorsal view of an embryo at stage 15. *Cav1L* expression is visible in the notochord and in a punctuated pattern in the skin. (**C**) Stage 18 embryo showing the same expression pattern as described in B. *Cav1L* is strongly expressed in the epidermis of a stage 20 embryo (**D**), stage 23 embryo (**E**), stage 26 embryo (**F**) and stage 28 embryo (**G**). (**H**) *Cav1* expression is visible in the notochord, epidermis and aortic arches of a stage 37 embryo. (**I**) *Cav1* is expressed in the notochord and the cardio-vasculature of the tail (dlav, pcv, isv) of a stage 41 embryo. (**I’**) Magnification of the embryo shown in I. *Cav1* is expressed in the lung, lymph heart and aortic arches. *Aa* aortic arches, *Dlav* dorsal longitudinal anastomosis vessel, *Isv/Isa* intersomitic vessels/artery, *lh* lymph heart, *lg* l ung, *nc* notochord, *Pcv* posterior cardinal vein.

In order to analyze how Cav1L affects muscle development and ultimately swimming behavior, we characterized the expression profile of *cav1L* during *Xenopus laevis* embryogenesis. RT-PCR showed that *cav1L* is maternally expressed at low levels in oocytes and early cleavage stages, while its expression increased at neurula and tadpole stages (supplementary Fig. S3A). Using in situ hybridization we detected *cav1L* expression at neurula stage 12.5—20 in the notochord and the neural plate (Fig. 2A–D, supplementary Fig S3B-D) as well as in the epidermis during neurula and tailbud stages (Fig. 2B–G). At late tailbud stages *cav1L* is expressed in the notochord, the lung, the heart as well as the vasculature of the branchial arches (aortic arches) and the tail (Fig. 2H–I’, supplementary Fig S3E-K). No specific staining was observed using a *cav1* sense probe (supplementary Fig S3L-O’).

**Figure 3:**
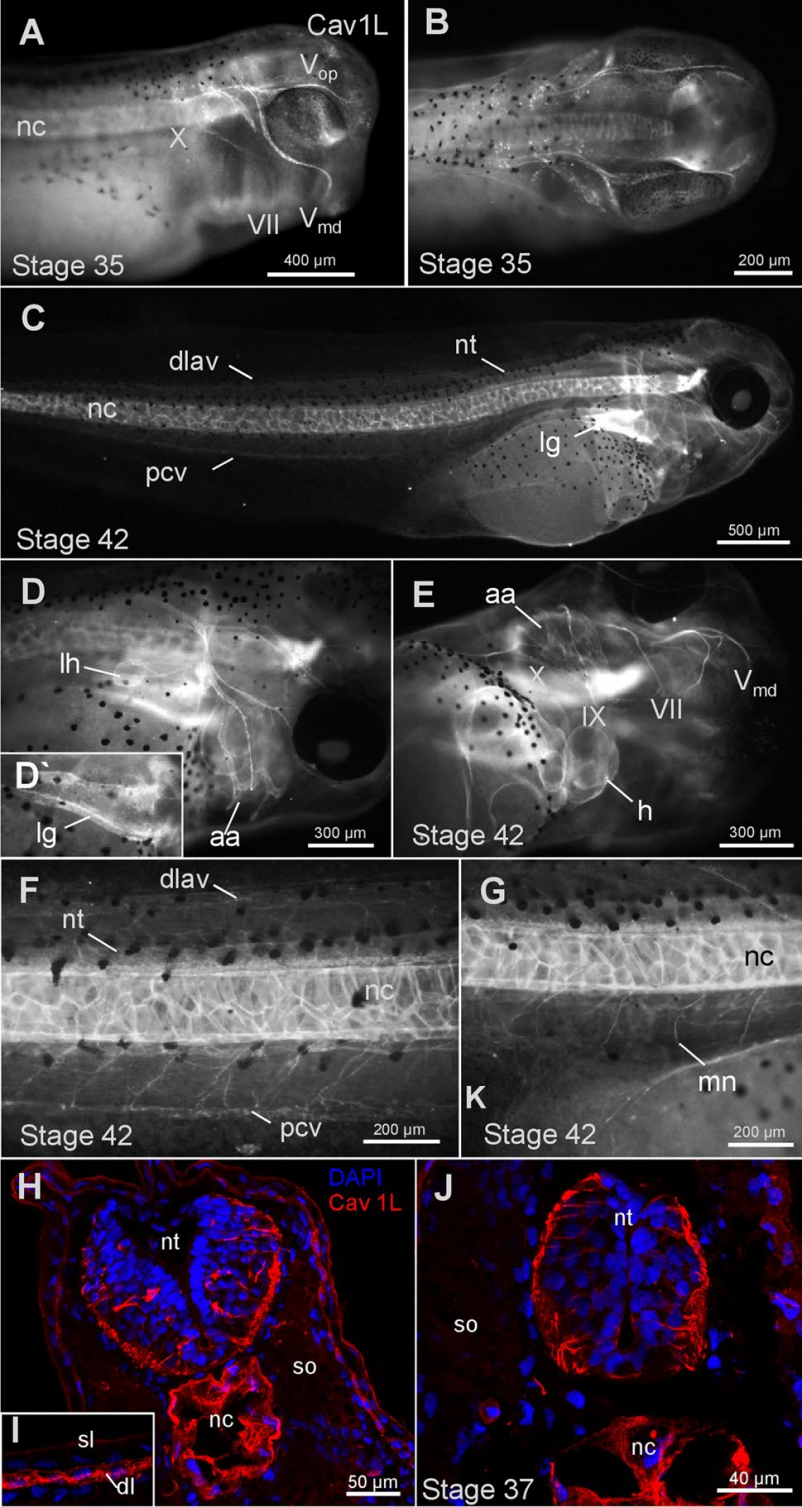
Cav1 is expressed in the nervous system during *Xenopus* development. (**A**) Immunostaining of a stage 35 embryo showing Cav1 protein expression in the notochord and the cranial nerves. (**B**) Dorsal view of a stage 35 embryos. Cav1 expression is visible in the cell bodies of the cranial nerve located in the brain. (**C**) Stage 42 embryo. (**D**) Higher magnification of a stage 42 embryo. Cav1 is expressed in the cranial nerves, aortic arches and the lymph heart. (**D’**) Image focusing on the Cav1 expression in the lung of the embryo shown in (**F**). (**E**) Ventral view of the embryo shown in (**D**). Cav1 staining is visible in the heart, aortic arches and cranial nerves. (**F**) Tail of a stage 42 embryos showing Cav1 expression in the cardio vasculature and neural tube. (**G**) Tail of a stage 42 embryo showing Cav1 expression in the notochord and motor neurons (**H**) Transverse section of the hindbrain of a stage 37 embryo showing Cav1 expression in red and DAPI staining in blue. Cav1 is expressed in neurons and the notochord. (**I**) Magnification of the epidermis of a stage 37 embryo. Cav1 is expressed in the deep layer of the epidermis. (**J**) Transverse section of the neural tube of a stage 37 embryo showing Cav1 expression in the neural tube and notochord. *Aa* aortic arches, *Dlav* dorsal longitudinal anastomosis vessel, *ep* epidermis, *h* heart, *il* inner layer of the epidermis, *Isv/Isa* intersomitic vessels/artery, *lh* lymph heart, *lg* lung, *mn* motor neurons, *nc* notochord, *nt* neural tube, *Pcv* posterior cardinal vain, *so* somites, *sl* sensory layer of the epidermis, *VII* facial nerve, *Vop* ophthalmic trigeminal ganglion, *Vmd* mandibular trigeminal ganglion, *IX* glossopharyngeal nerve, *X* vagus nerve.

Complementing the in situ data, Cav1 protein expression was analyzed by immunofluorescence using an antibody detecting both isoforms α and β (Fig. 3). Cav1 protein was detected in the notochord (Fig. 3A,C,F,G), the lung (Fig. 3C,D), the heart and the vasculature of the branchial arches and the tail (Fig. 3E,F) and the epidermis (Fig. 3I). Interestingly, Cav1 protein is also expressed in the nervous system, including cranial nerves and motor neurons (Fig. 3A,E,G). Transverse sections of stage 37 embryos confirmed that Cav1 is expressed in neuronal cells of the spinal cord, the notochord as well as the epidermis (Fig. 3H,J). Taken together, Cav1 expression (protein or RNA) was not detected in the musculature, neither by in situ hybridization nor by immunofluorescence staining. Thus, Cav1 likely functions in muscle innervation rather than directly in the musculature.

**Figure 4:**
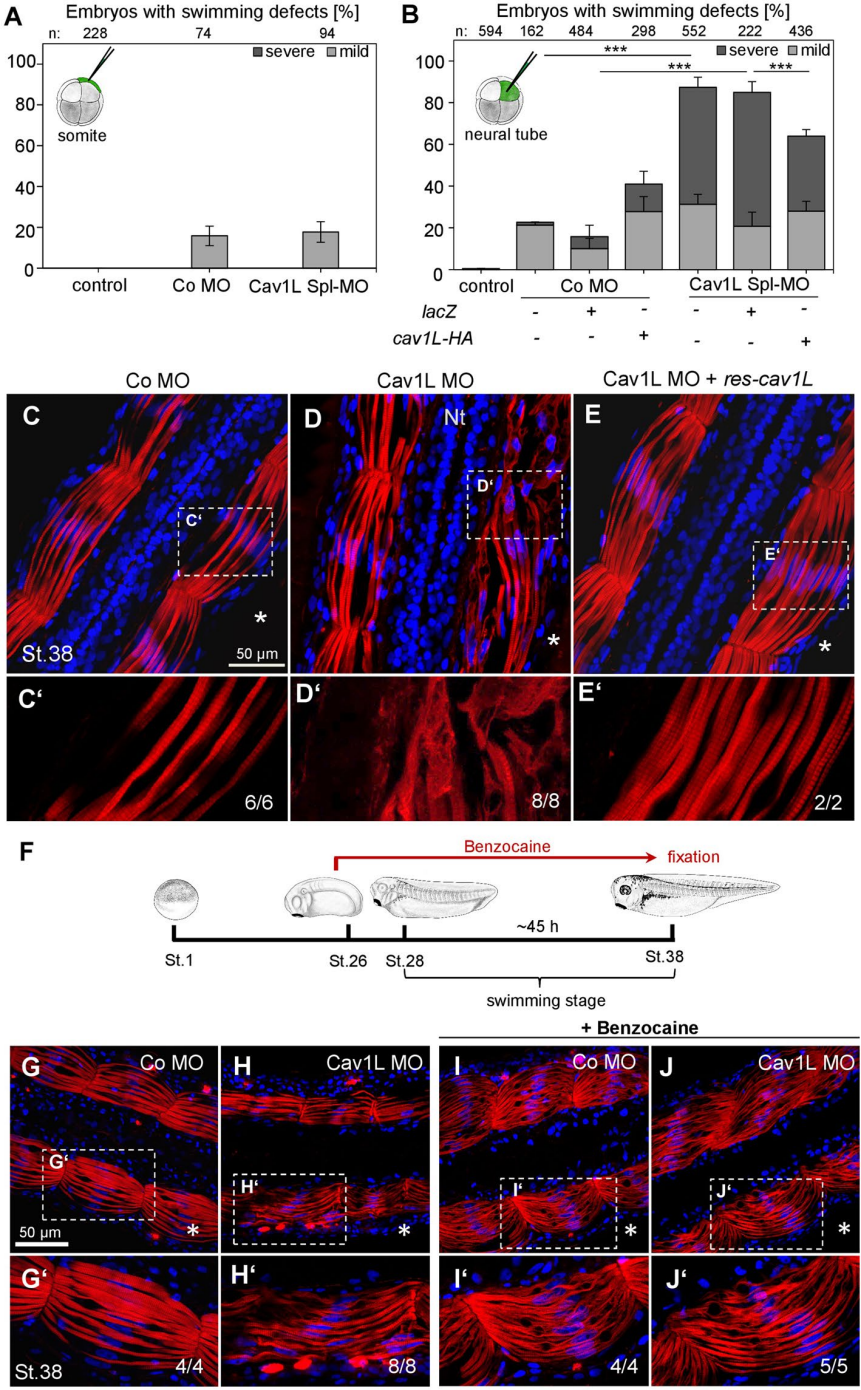
Loss-of-function of Cav1L in neural tissue or Benzocaine-treatment causes muscular disorganization and swimming defects. (**A,B**) 10 ng MO alone or in combination with 100 pg *cav1L-HA* RNA were targeted to the somites (**A**) or the neural tube (**B**) at the 8-cell stage and the percentage of swimming defects (three independent experiments) were analyzed at stage 38. *LacZ* RNA injection was used to control for RNA toxicity or unspecific rescue effects. Data from at least three experiments are presented as the mean ± s.e.m.. ***p-value ≤ 0.001 (one-way ANOVA comparing the total number of swimming defects). (**C**–**E**) Muscle morphology of neural-injected embryos were analyzed by sectioning and Phalloidin staining. Asterisks indicate the injected side. (**C**) Normal actin organization in a control embryo. (**D**) Highly disrupted actin organization on the injected side of Cav1-morphant embryo. (**E**) Co-expression of *res-cav1L* RNA in neural tissue restores actin organization of Cav1L-morphant muscle cells. (**C’,D’,E’**) Higher magnification of the boxed areas shown in (**C**–**E**). (**F**) Embryos were injected unilaterally with 20 ng MO and cultivated until stage 26 when Benzocaine was added to the medium. Embryos were fixed at stage 38, sectioned longitudinally and stained with Phalloidin. Asterisks in (**G**–**J**) indicate the injected side. (**G**) Normal muscle morphology of a control embryo. (**H**) Loss-of-function of Cav1L disrupts somitic actin fiber organization. (**I,J**) Benzocaine-treated embryos. The absence of neural activity caused by Benzocaine-treatment results in disorganized actin fibers in controls (**I**) as well as Cav1L-morphant embryos (**J**). (**G’,H’,I’,J’**) Higher magnification of the boxed areas shown in (**G**–**J**).

To further dissect if Cav1L function is required in neural versus mesodermal tissue targeted injections were performed. Swimming behavior was analyzed at stage 38 whereby we distinguished either between a circling movement (severe defect) or a slow forward movement (mild defect). Indeed, mesodermal injections did only cause few swimming defects (Fig. 4A, supplementary Fig. S4A). In contrast, targeted Morpholino injection into the neural tissue resulted in a high percentage of swimming defects. These defects could be significantly improved upon co-injection of *cav1L* RNA (Fig. 4B, supplementary Fig. S4B). This was also confirmed by analyzing the sarcomeric structure of these embryos using phalloidin staining (Fig. 4C–E). In contrast to Co MO-injected embryos (Fig. 4C), Cav1L morphants showed a disrupted actin network on the injected side (Fig. 4D), which was restored upon co-injection of a wild-type *cav1L* construct lacking the Morpholino binding site (*res-cav1L,* Fig. 4E). In contrast, co-injection of *lacZ* RNA did not improve the swimming behavior of the morphant embryos (Fig. 4B). Thus, these data indicate that Cav1 function is required in the neural circuits controlling muscular integrity.

### Inhibition of voltage-gated sodium channels mimics the Cav1L loss-of-function phenotype

*Cav1L* is expressed in the nervous system, but it is also required for muscular function. Since it is well-known that defects in neuromuscular activity can lead to muscular atrophy^38–40^, we asked if the muscular phenotype may be caused by defects in muscular innervation. To this end we treated embryos—injected with the Cav1L MO or Co MO—prior to the onset of the swimming stage with Benzocaine, an inhibitor of voltage-gated sodium channels (Fig. 4F). This treatment blocks the transmission of an action potential from the motor neurons to the muscle cells mimicking a denervated muscle. Embryos were cultured in the anesthetic until stage 38 when they were stained with Phalloidin to analyze muscle morphology. Actin filaments were correctly aligned in untreated controls, but Cav1L-morphant embryos displayed disorganized actin filaments (Fig. 4G,H). Benzocaine-treated embryos—including the uninjected controls—showed the same muscular defects as untreated Cav1L morphants (Fig. 4I,J). Interestingly, the severity of the morphant phenotype was not increased by Benzocaine-treatment. Thus, these data suggest that the observed muscle phenotype is likely caused by defects in innervation.

### Cav1L loss-of-function causes defects in axon outgrowth and neuronal morphology by affecting Rho GTPases

**Figure 5:**
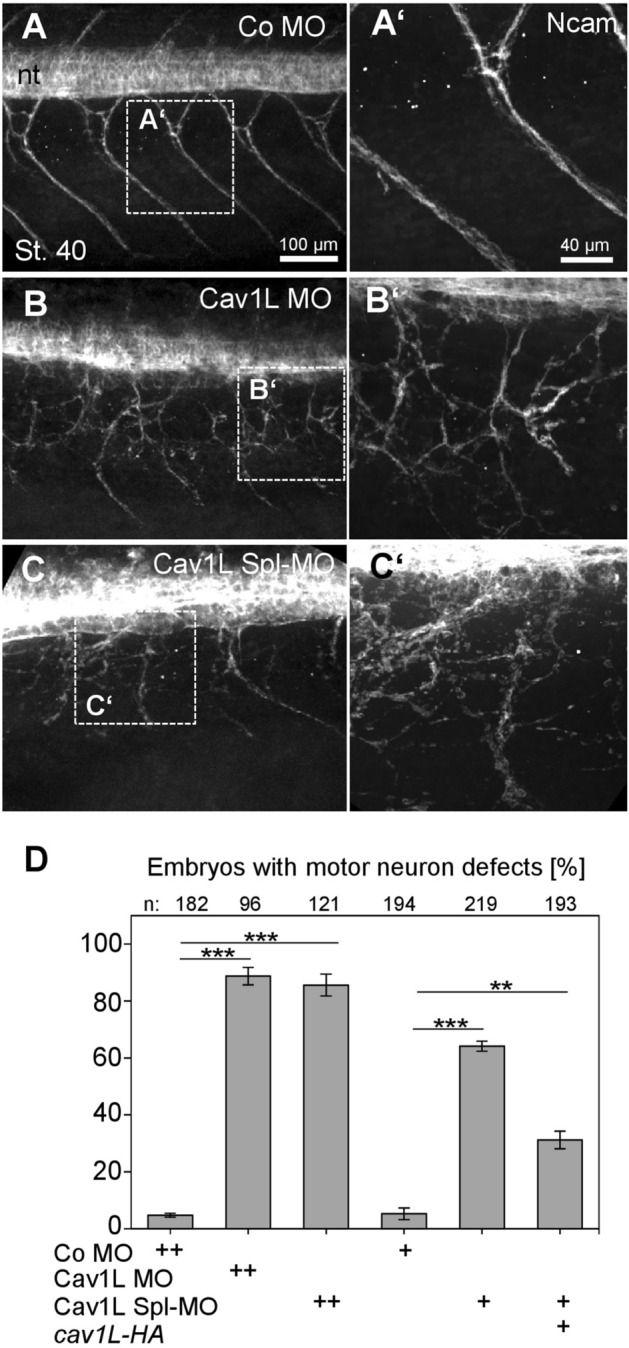
Loss of Cav1L affects motor neuron morphology. (**A**–**D)** Embryos, injected unilaterally with 20 ng MO at the two-cell stage, were immunostained using the neuronal surface marker Ncam. (**A**) Motor neurons display a characteristic chevron-shaped pattern in controls; nt = neural tube. (**B,C**) Motor neuron outgrowth and pathfinding is severely impaired in Cav1L morphants. (**A’,B’,C’**) Higher magnification of the dashed areas shown in (**A**–**C**). (**D**) Percentages of motor neuron outgrowth defects of Morpholino-injected embryos (20 ng, + +) and embryos injected unilaterally with 10 ng MO (+) in combination with 200 pg *cav1L-HA.* Co-expression of *cav1L-HA* partially rescues motor neuron defects. Graph in (**D**) present data as the mean ± s.e.m. from at least three experiments. Number of analyzed embryos is indicated for each column. **p-value ≤ 0.01 ***p-value ≤ 0.001 (one-way ANOVA).

As Cav1L is expressed in motor neurons we next investigated if loss-of-function of Cav1L also affects *Xenopus* nerve morphology*.* To this end, we analyzed motor neuron morphology by immunostaining using an N-CAM antibody on embryos injected unilaterally with 20 ng MO. In control embryos axons from motor neurons innervated the musculature in a characteristic chevron-shaped pattern (Fig. 5A). In contrast, motor neurons were severely affected by loss of Cav1L function; although they were still able to form axons, they randomly projected their axons in the periphery and did not follow the boundaries of the somitic muscles (Fig. 5B–D). Furthermore, injection of the Cav1L Spl-MO, which is also more effective in blocking Cav1L expression compared to the translation blocking Cav1L MO (Fig. 1B), showed both severe pathfinding (Fig. 5C) and outgrowth defects (supplementary Fig. S5B). In order to analyze if the neuronal phenotype is specific for Cav1L loss-of-function, rescue experiments were performed*.* While the injection of the Cav1L Spl-MO caused motor neuron outgrowth and pathfinding defects, co-expression of *cav1L-HA* RNA significantly rescued these defects (Fig. 5D, supplementary Fig. S5C), indicating that the observed neuronal abnormalities are indeed specific to Cav1L loss-of-function.

To examine if Cav1L also affects early neuronal patterning we analyzed if Cav1L loss-of-function affects the expression of the neuronal marker *n-tubulin* (neuron specific class IIβ tubulin) at early neurula stages (supplementary Fig. S6). Control embryos, as well as the majority of the Cav1L-morphant embryos showed normal *n-tubulin* expression in all four domains, indicating that loss-of-function of Cav1L has no impact on early neurogenesis and neuronal patterning in *Xenopus* embryos (supplementary Fig. S6). Furthermore, *shh* expression in the floorplate (supplementary Fig. 7) and *BMP4* expression in the roofplate (data not shown) was also not affected suggesting that dorsal/ventral patterning of the neural tube is not disturbed in Cav1L morphants. Thus, the muscular defects of Cav1L-morphant embryos are likely caused by impaired muscular innervation due to defects in axonal outgrowth and pathfinding of motor neurons.

**Figure 6:**
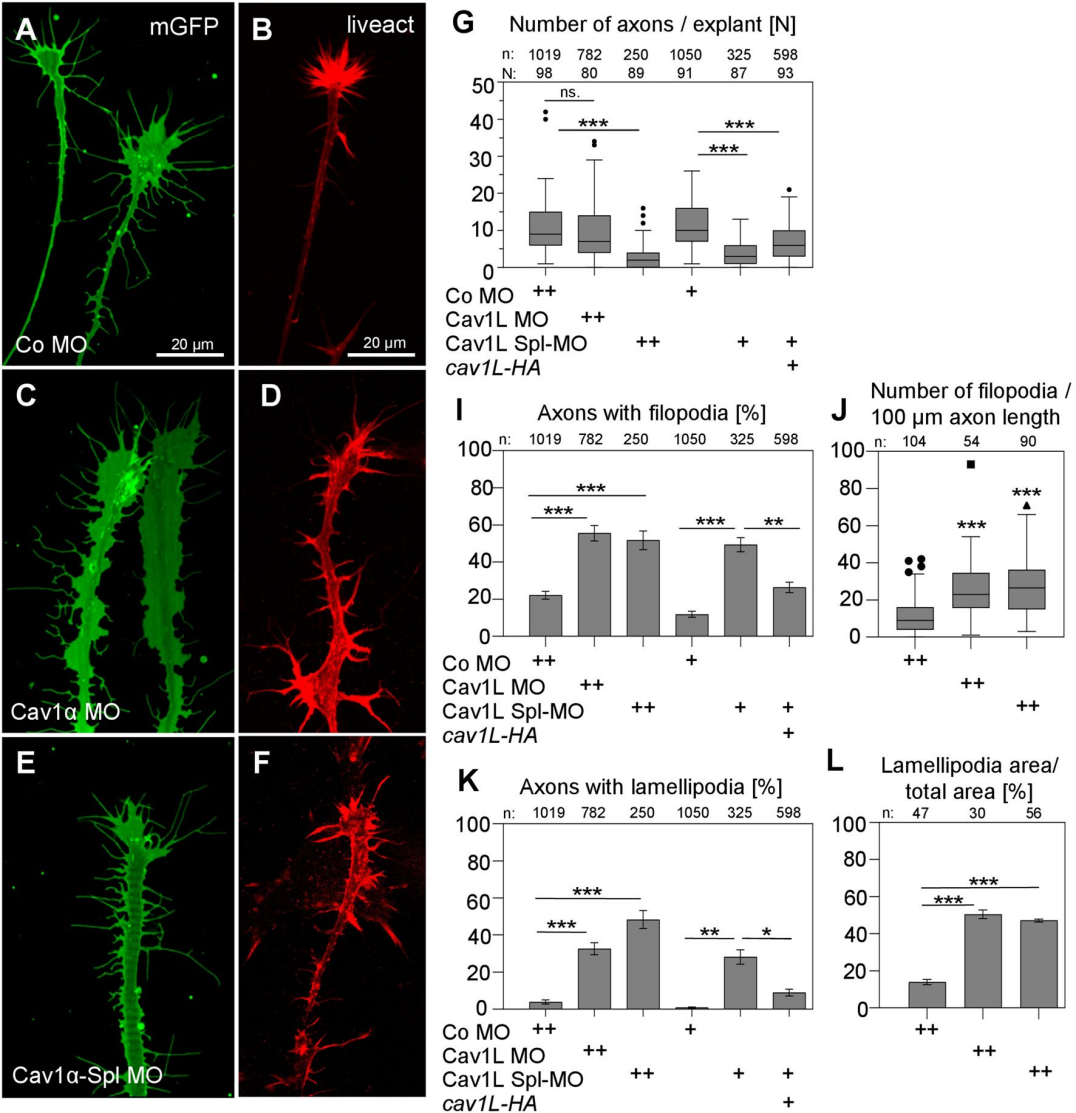
Cav1L knockdown affects axonal growth and morphology in vitro. (**A**–**N)** Loss-of-function of Cav1L increases filopodia as well as lamellipodia formation in cultured spinal cord neurons, isolated from embryos that were injected with either 12 ng (++) or 10 ng (+) Morpholino in both blastomeres at the two-cell stage. Actin staining is shown in (**D**) (Co MO), (**E**) (Cav1L MO) and (**F**) (Cav1L Spl-MO). (**A,B**) Control axons show typical actin-positive growth cones. (**C–F**) Filopodia- and lamellipodia-like actin-positive structures in Cav1L-morphant neurons. (**G**) Box plot showing the number of axons (n) per explant (N) (Tukey Box plot with whiskers with maximum 1.5 IQR (Mann–Whitney test)). Co-expression of 10 pg Cav1L Spl-MO in combination with 200 pg *cav1L-HA* RNA significantly improved axonal outgrowth. (**I**) Percentage of axons with increased filopodia-like structures. Co-expression of 200 pg *cav1L-HA* RNA significantly decreased the number of axons with filopodia. (**J**) Number of filopodia per 100 µm axon length. (**K**) Percentage of axons with lamellipodia. Co-expression of 200 pg *cav1L-HA* significantly decreased the number of axons with lamellipodia. (**L**) Percentage of lamellipodia area in relation to total axon area. Data in (**I,K,L**) are mean ± s.e.m. from at least three experiments (Student’s t-test). Number of analyzed axons (n) are indicated for each column. * p-value ≤ 0.05; **p-value ≤ 0.01; ***p-value ≤ 0.001.

To further characterize axon outgrowth, neural tubes of stage 20 embryos, injected with 12 ng Morpholino and the lineage tracer GFP, were explanted and analyzed after one day of incubation. Consistent with the in vivo data Cav1L-morphant axons showed defects in outgrowth and morphology (Fig. 6). Again, we noted that the defects caused by the Cav1L Spl-MO—which is also more potent in blocking Cav1L expression compared to the translation blocking Cav1L MO—were more severe and fewer axons extended in vitro (Fig. 6G). This effect was specific and could be rescued by co-expression of *cav1L-HA* RNA (Fig. 6G). Furthermore, while control neurons within the explants extended long axons with the typical actin-rich growth cone (Fig. 6A,B), a significant larger number of axons from Cav1L-morphants displayed an increase in actin-rich filopodia- and lamellipodia-like structures (Fig. 6C–F,I–L). The number of axons with filopodia, as well as the number of filopodia per axon was significantly increased (Fig. 6I,J). Likewise, the number of axons with lamellipodia and the lamellipodia per axon was significantly increased in morphants compared to controls (Fig. 6K,L). The defects in axon morphology were specific for Cav1L loss-of-function and could be rescued by co-injection of *cav1L-HA* RNA (Fig. 6I,K). Thus, Cav1L is not only expressed in motor neurons but also required for axonal outgrowth and morphology in vivo as well as in vitro.

**Figure 7:**
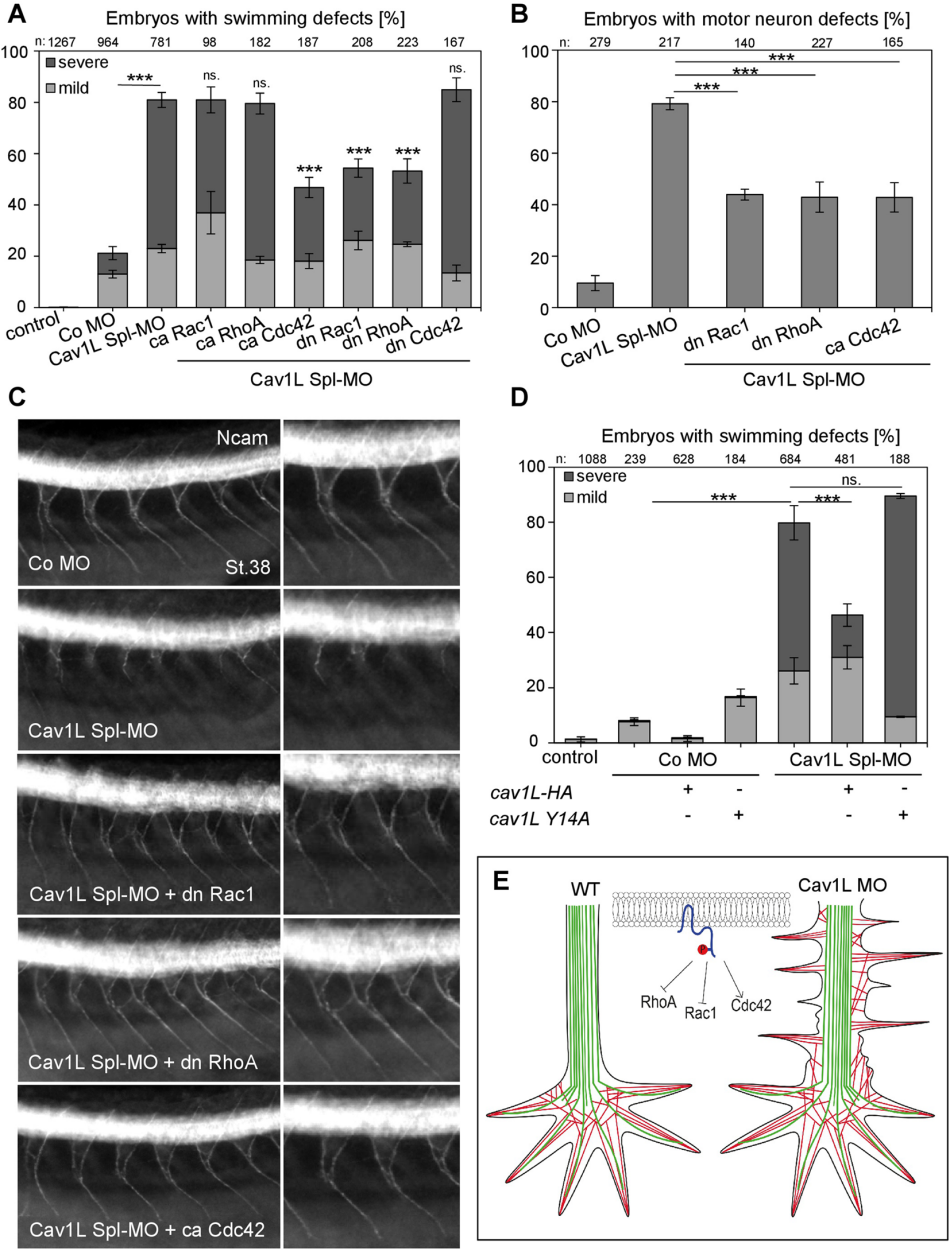
Y14 phosphorylation of Cav1L is necessary for Rho GTPases dependent axonal outgrowth and pathfinding of motor neurons. (**A,B**) Co-injection of 10 pg ca Cdc42, dn RhoA or dn Rac1 with 10 ng Cav1L Spl-MO in neural tissue significantly improved swimming defect (**A**) and motor neuron outgrowth (**B**) caused by Cav1L loss-of-function. Significances of the rescue experiments were calculated in comparison to the Cav1L Spl-MO injected embryos. (**C**) Motor neuron outgrowth was analyzed by Ncam immunostaining, injected constructs are indicated. (**D**) Overexpression of 200 pg *cav1L Y14A* RNA in Cav1L-morphant (10 ng) embryos did not rescue swimming defects, while 200 pg of wild-type *cav1L* significantly improved the morphant phenotype, when injected into one blastomere of the two-cell stage. All graphs (**B**–**D**) present data from at least three experiments as the mean ± s.e.m. ***p-value ≤ 0.001 (one-way ANOVA comparing the total number of swimming defects). (**E**) Model of Cav1L function in motor neuron outgrowth. Tyrosine phosphorylated Cav1L (blue, with red Y14 phosphorylation) affects Rho GTPases. The morphology of a wild-type (WT) and Cav1L-morphant motor neuron is shown; microtubules (green) and actin bundles (red) are shown.

As Rho GTPases are known regulators of lamellipodia and filopodia formation during axonal growth and guidance^41,42^ and as Cav1 is known to modulate the activity of RhoA and Rac1 in mouse embryonic fibroblasts^43,44^ we analyzed if constitutive active or dominant negative RhoA, Rac1 and Cdc42 are able to rescue the morphant phenotypes if injected into neural tissue. Indeed, dominant negative Rac1 as well as RhoA, but not the constitutive active mutants could partially rescue the swimming behavior as well as the neuronal defects caused by loss of Cav1L function (Fig. 7A–C). Conversely, constitutive active but not dominant negative Cdc42 partially rescued the aberrant swimming behavior and neuronal defects (Fig. 7A–C). This indicates that Cav1L affects motor neuron morphology by suppressing RhoA and Rac1 activity and supporting Cdc42 activity.

### The Cav1 Y14 phosphorylation site is required for the function of Cav1L in the locomotor system of *Xenopus* tadpoles

It has been shown that Src-dependent tyrosine (Y14) phosphorylation of Cav1 plays an important role in the regulation of RhoA and Rac1/Cdc42 activity^43–45^. In order to determine if this modification is required for Cav1L function in the nervous system of *Xenopus* tadpoles, we performed rescue studies with a non-phosphorylatable Cav1L mutant (CavY14A). Morpholinos in combination with *cavY14A* RNA were unilaterally injected at the two-cell stage and swimming behavior was analyzed at tadpole stages. The phosphorylation mutant was not able to rescue swimming defects if co-expressed with the Cav1L Spl-MO, while full-length *cav1L* partially rescued (Fig. 7D). This indicates that the Y14 phosphorylation is required for Cav1L function and possibly the regulation of Rho GTPases in the locomotor system of *Xenopus laevis*.

## Discussion

A well-established neuromuscular communication is important for muscle development and integrity and loss of neural connectivity causes severe muscle atrophy^46–50^. Here we report a novel function of Cav1 in the development of the *Xenopus* neuromuscular system: Cav1L is expressed in motor neurons and required for muscular innervation. Loss of function causes impaired muscular integrity and paralysis of morphant embryos. As we find that axonal outgrowth and morphology are severely affected in morphants, while the induction of primary neurons per se is not compromised, we suggest that Cav1L function in neural development is likely limited to post-induction stages. Our data suggest a model whereby Cav1L functions in *Xenopus* motor neuron outgrowth by regulating lamellipodia and filopodia formation of axons (Fig. 7E). In the wild-type situation this is likely mediated by supporting Cdc42 activity and suppressing RhoA and Rac1 activity. In Cav1L morphant axons, lamellipodia and filopodia are not retracted and the growing axons display an increase in protrusions. Further Cav1L function likely requires the tyrosine 14 phosphorylation site, as this phosphomutant (Y14A) was not able to rescue the morphant swimming defects. Thus, these data indicate a novel function of Cav1L in in vivo development of *Xenopus* motor neurons and reveal an indirect role in muscular function and embryonic mobility.

Coordinated regulation of the small Rho GTPases Rac1, RhoA, and Cdc42 in lamellipodia and filopodia formation of the growth cone is essential for controlled growth and navigation of axons^41,51^. Furthermore, it has been shown that Cav1 plays an important role for the fine tuning of Rho GTPase activity during cell-migration in different cellular systems^43–45^. Mouse embryonic fibroblasts from Cav1 knockout mice display defects in cell polarity and migration, caused by misregulation of Src, RhoA, Rac1 and Cdc42 activity^43^. For example it has also been shown that Cav1 regulates the ubiquitination and degradation of active Rac1 at focal adhesion sites^44^. Further it was suggested that Cav1 activates RhoA by inactivation of Src: tyrosine (Y14) phosphorylated Cav1 binds to and activates Csk, which subsequently phosphorylates and inactivates Src in a negative feedback loop^43,52,53^. Similar mechanisms are likely also acting in axonal development as inhibition of Cav1 Y14 phosphorylation inhibited Rac1/Cdc42-mediated axonal growth in human neurons derived from induced pluripotent stem cells^54^. It is currently unclear how Cav1L affects Rho GTPases in *Xenopus* motor neurons, however, in respect to the studies on isolated neurons, a mechanism affecting the activity of these GTPases seems likely.

Evidence for a neuronal function of Cav1 has already been demonstrated by loss of function studies in mice. Cav1 knockout mice display distinct traits associated with progressive neurodegeneration such as deficits in motor coordination, gait abnormalities (shorter stride length), muscle weakness as well as a clasping and spinning phenotype^29^. Additionally, they also show behavioral changes associated with cholinergic dysfunction, characterized by impaired spatial memory, increased anxiety as well as reduced exploratory behavior in a new environment^29,32^. It remains unclear if *Xenopus* Cav1L morphants would also show neurodegenerative defects, as our Morpholino-mediated approach allowed us only to analyze its role in early neurodevelopment. In vivo as well as overexpression studies of mouse and human hippocampal cell cultures have shown that Cav1 positively regulates neuronal plasticity and neuronal intracellular signaling by recruiting neurotransmitters and neurotrophic factors to synaptic membrane lipid rafts^54–57^. Membrane lipid rafts are especially important for pro-survival and pro-growth receptor signaling in neuronal cells, since receptors and proteins required for synaptic communication mainly localize in these membrane domains^58^. Further, membrane lipid rafts at the leading edge of the growth cone are important reservoirs for signaling molecules, such as Rho GTPases, integrins and N-cadherins, which are essential for actin dynamics and adhesion^59–61^. Cav1 has been shown to modulate the nanoscale lipid organization of specialized membrane lipid raft domains by regulating the expression of metabolic proteins, such as Ppap2A (Lpp1), B3GNT5 and Siat9 (GM3 synthase), which are involved in glycosphingolipid, sphingolipid as well as ganglioside biosynthesis^62–64^. Ganglioside expression is tightly regulated during the development of the peripheral and central nervous system and misexpressions of these lipids are associated with progressive neurodegeneration in mice and humans^65–67^. Interestingly, mice lacking Siat9 and thus the expression of complex gangliosides, displays similar neurological deficiencies as Cav1 knockout mice^68,69^. Thus, the neurological defects caused by the loss of Cav1 likely result from a disturbed membrane lipid raft composition and the resulting mislocalization of signaling molecules regulating axonal biology.

Another mechanism by which Cav1 could regulate axonal growth is by affecting voltage-gated sodium channels and thereby regulating neuronal activity. In cardiac cells, it has been shown that the voltage-dependent sodium channel Na_V_1.5 localizes to caveolae membrane domains^70^. Interestingly, the inhibition of voltage-gated sodium channels, such as Na_V_1.9, by either knockout or Tetrodotoxin (TTX) treatment, impairs axonal growth of cultured mouse motor neurons^71^. These neurons display shorter motor axons as well as reduced spontaneous Ca^2+^ transients, which are required for axonal growth and the establishment of synaptic connections^71–75^. It has been suggested that the activity of Na_V_1.9 and consequently Na_V_1.9-dependent spontaneous Ca^2+^ transients are regulated by activated TrkB in neuronal cells^71,76,77^. Egawa et al. were able to show that Cav1 recruits receptor tyrosine kinase B (TrkB) to membrane lipid rafts in vivo and improves TrkB signaling in vitro in hippocampal neurons^36,55,57^. Although a direct link of Cav1 to the regulation of neuronal activity has not yet been described, Cav1 might have a function in this process by recruiting TrkB to MRLs in neuronal cells, which in turns modulates the activity of voltage-gated sodium channels located in these membrane domains.

A question that remains is if the swimming defects of Cav1L morphants are also impacted by defects in notochord development. Indeed, *Xenopus* Cav1L is highly expressed in the notochord, which serves as important structural element supporting locomotion in free-swimming larvae. Caveolae are essential structures protecting the notochord against mechanical stress^78–80^ and their depletion causes mechanical-induced collapse of notochord cells^78,81^. In zebrafish embryos, it has been shown that defects in notochord development can affect both the axial skeleton as well as muscular innervation^82,83^. Interestingly, loss of *cavin1b* function in the zebrafish notochord, which abolishes caveolae-formation, affects swimming behavior^81^. However, in contrast to *Xenopus* Cav1L-morphant embryos, which were completely paralyzed when Cav1L MO was injected into both blastomeres of a two-cell stage embryo, zebrafish depleted of caveolae only swam shorter distances compared to wild type embryos, while overall swimming behavior was not affected^81^. In support of these findings we also noted that loss of function of *Xenopus* cavin1 does not affect the swimming behavior of *Xenopus* embryos (data not shown). Thus, the observed muscular defects of *Xenopus* Cav1L morphants are likely not caused by defects in notochord development. Moreover, single loss of function of either zebrafish *cav1* or *cav3* was not sufficient to induce mechanical induced lesions in the notochord suggesting that other caveolin proteins likely compensate for the loss of a single caveolin isoform^78,81^. We expect a similar situation in *Xenopus*, where different caveolin paralogs are expressed in the notochord^84^. Interestingly, we note that targeted injection of Cav1L morpholino to one side of the embryo caused paralysis only on the injected side; however, notochord cells intercalate during development, thus irrespective of the site of injection, we would expect both sides to be affected. Thus, in combination with our in vitro analysis showing axonal outgrowth and morphology defects these data argue for a direct function of Cav1L in neuromuscular development.

## Material and methods

### Constructs

For knockdown studies the following Morpholinos were generated by Gene Tools, LLC (Philomath, OR, USA): Cav1L translation blocking Morpholino (Cav1L MO^85^), 5′-CATCTATGTATTTGCCACCAGACAT-3′); Cav1L Splice-blocking Morpholino (Cav1L Spl-MO, 5′-CAGCGCCCAGATCATACAGCCTTAC-3′); standard control Morpholino (Co MO, 5′-CCTCTTACCTCAGTTACAATTTATA-3′); The Splice MO was designed to specifically bind the Exon2-Intron2 boundary of the endogenous *Cav1L* pre-mRNA (For Morpholino design see Supplementary Fig. 1). Both Cav1 Morpholinos target the *Xenopus laevis* Cav1L paralog.

For the detection of endogenous *Cav1L* mRNA, sense and antisense RNAs were amplified from xcaveolin-1α pCMV-Sport6 (RZPD, catalogue number IRBMp990B0725D). The caveolin1L-rescue construct (res-cav1L-HA) was cloned from xcaveolin1-HA pCS2+^85^ using the forward primer 5′-ATGCTAGCATGGAAGAGGGTGTTCTCTACAC-3′ and the reverse primer 5′-ATGCTAGCGAATCGATGGGATCCTGCAAA-3′ to generate a truncated construct lacking the Morpholino binding side. The Cav1L Y14 phosphorylation mutant was cloned by site-directed mutagenesis from xcaveolin1-HA pCS2 + using the forward primer 5′- TGAAGAGGGTGTTCTCGCCACCACGCCGGTCATC-3′ and the reverse primer 5′ GATGACCGGCGTGGTGGCGAGAACACCCTCTTCA-3’.

For overexpression analysis the following plasmids were used: lacZ pCS2+^86^ mGFP^87^, xcaveolin1-HA pCS2+^85^, ca Cdc42 V12 pCDNA3.1^88^, dn Cdc42 N17 pCS2+^89^; the constructs ca RhoA (V12/V14) N17 pCS2 + , dn RhoA N19 pCS2 + , ca Rac1 V12 pCS2 + and dn Rac1 (N17) pCS2 + were cloned from their original pcDNA3.1 vectors^88,90^ into the pCS2 + vector. For microinjection RNA was synthesized using the mMessage Machine Kit (Ambion, Life Technologies) according to the manufacturer’s instructions.

### *Xenopus* injection and phenotypic analysis

*Xenopus laevis* embryos were obtained by i*n vitro* fertilization and staged according to Nieuwkoop and Faber^91^. All procedures were performed according to the German animal use and care law (Tierschutzgesetz) and approved by the German state administration Hesse (Regierungspräsidium Giessen). Microinjections were performed either into one blastomeres of two-cell stage embryos with an injection volume of 10 nl or into one blastomere of eight-cell stage embryos using an injection volume of 5 nl, respectively. To specifically target the musculature, embryos were injected in the dorsal blastomere of the vegetal hemispheres; to target the brain and spinal cord, embryos were injected into the dorsal blastomere of the animal hemispheres. For western blot analysis embryos were injected at the one-cell stage. *mGFP* RNA was always co-injected as lineage-tracer with a concentration of 75–80 pg.

Swimming behavior was analyzed by stimulating the escape response of the embryos in response to a touch stimulus. Swimming behavior was counted as normal, when the embryos were able to move their tail in both directions. Embryos were defined as having a mild defect, when they were still able to swim slowly, however their tail movement was visible impaired. Embryos with a severe swimming defect were completely paralyzed on the injected site and only moved in circles or were unable to swim.

### RT-PCR

For the verification of the Splice-blocking MO as well as the time course analysis of *cav1L* expression, total RNA was isolated from *Xenopus* embryos. For Morpholino verification, stage 20 embryos and for the time course analysis, embryos of different stages were collected and frozen in liquid nitrogen. RNA isolation was performed using the GE Healthcare illustra RNAspin Mini Isolation Kit following the manufacturer's instructions and cDNA was synthetized accordingly; 1 ng–5 µg RNA was incubated together with 0.2 μg random hexamer Primer (Thermo Fisher Scientific), 1 mM dNTP Mix (Thermo Fisher Scientific) in a final volume of 10 µl for 5 min at 65 °C. Afterwards, 20 units of MuLV Reverse transcritprase (Thermo Fischer Scientific), 2 µl of 5 × RT Reaction Buffer (Thermo Fisher Scientific) and 20 units of RNaseOUT (Invitrogen), adjusted to a final volume of 10 µl using nuclease free water, were added to the mix. The mix was incubated at 25 °C for 5 min and subsequently at 42 °C for 1 h. Reaction was terminated by incubation for 20 min at 65 °C. For amplification of the full-length Cav1L sequence a 5′UTR primer (5′-CCAGCAACTGAAGGACAGC-3′) and a reverse Primer (5′-GAATCGATGGGATCCTGCAAA-3′) were designed. For the time course analysis Cav1L forward primer (5′-TGGCAGATGATATGCTGACTGA-3′) and reverse primer (5′ CCCGGCTAAGGAACTGAATCT-3′) were used. PCR products were imaged using the Odyssey Fc Imaging System (LI-COR Bioscience).

### Preparation of protein lysates and Western blotting

Protein extracts were prepared by lysis of injected *Xenopus* embryos with insulin syringes in 10 µl/embryo Lysis buffer (50 mM Tris–HCl pH 7.5, 150 mM NaCl, 0.5% NP40 (v/V), 0.1% SDS, EDTA-free 1 × CompleteProteaseInhibitor (Roche). Lysates were centrifuged for 15 min by 16.000*g* at 4 °C and supernatants were transferred to a fresh Eppendorf tube. Protein lysates were diluted 1:5 with 6 × Laemmli loading buffer (350 mM Tris–HCl pH 6.8, 9.3% Dithiothreitol, 30% (v/v) glycerol, 10% SDS, 0.02% Bromophenol Blue) denatured for 5 min at 95 °C and subsequently loaded to a 10 or 12% SDS-PAGE gel. Following separation, proteins were transferred to a nitrocellulose membrane (Whatman) by wet- or semi-dry blotting (Mini PROTEAN Tetra System; Trans-Blot-turbo Transfer System, BIO-RAD). The membrane was blocked in TBST-blocking buffer (50 mM Tris–HCl pH 7.5, 150 mM NaCl and 0.5% (v/v) Tween 20) containing 5% nonfat dried milk for at least 30 min. The following primary antibodies were used: anti-caveolin-1 (Abcam, ab2910, 1:300), anti-GAPDH (AM4300, Thermo Fisher Scientific, 1:5000). The antibodies were removed by washing three times for 10 min each in blocking solution and the secondary anti-mouse-HRP antibody (sc-516 102, Santa Cruz Biotechnology, 1:5000) was applied for 1 h at room temperature. Chemiluminescence was detected using SuperSignal West Dura Extended Duration Substrate (Thermo Fisher Scientific) and Odyssey Fc Imaging System (LI-COR Bioscience) and analyzed by Image Studio Software (LI-COR).

### Whole mount immunofluorescence staining and in situ hybridization

Embryos were fixed in ice-cold Dents (80% Methanol, 20% DMSO) over-night at 4 °C. The fixed embryos were washed twice in PBS and then photo-bleached in 2% H_2_O_2_ in 1 × PBS for 4–5 h under a light source. Embryos used for caveolin 1 expression analysis were not treated with H_2_O_2_. The embryos were rinsed in PBS-TD (0.137 M NaCl, 2.7 mM KCl, 10 mM Na_2_PO_4_, 1.8 mM KH_2_PO_4_, pH 7.4; 1% Triton X-100, 1% DMSO) and then washed twice in 50 mM sodium acetate buffer (pH 6) for 5 min each. Afterwards embryos were treated with 1 mg/ml bovine testicular hyaluronidase (Sigma, SLBH0986V) in 50 mM sodium acetate buffer (pH 6.0) for 45 min at room temperature. Embryos were rinsed again twice in PBS-TD and blocked for 4 h in PBS-TD containing 0.1 M glycine, 2% nonfat dried milk, and 5% FBS at room temperature and incubated with the first antibody in blocking solution over-night. The following primary antibodies were used: Ncam (DSHB, 4d,1:30), Caveolin 1 (BD Transduction Laboratories, 610406, 1:100), GFP (Abcam, 278239, 1:1000). After primary antibody reaction, embryos were washed in PBS-TD six times for one hour each and then incubated overnight with secondary antibodies: anti-mouse Alexa 594 (Invitrogen, A-11005, 1:400), anti-mouse Alexa 488 (Invitrogen, A11029, 1:400), anti-rabbit Alexa 488 (Invitrogen, A-21206, 1:400). Embryos were washed six times for one hour each in PBS-TD at room temperature and then re-fixated in Dents overnight.

For imaging, embryos were cleared in Benzyl-alcohol/Benzyl-Benzoate (Sigma Aldrich) (BA/BB; 1:2). Therefore, embryos were washed two times in 100% ethanol. For clearing embryos were incubated first in BA/BB for 10 min and then in fresh BA/BB for imaging. Embryos were imaged using the Zeiss Spinning Disc system (Axio Observer Z1 with a 25 × or 40 × water objective) or a fluorescence stereo-microscope (Leica, M165-FC).

For whole mount in situ hybridization embryos were fixed in MEMFA 3.7% formaldehyde, 0.1 M MOPS, 2 mM EGTA, 1 mM MgSO_4_) and analyzed according to standard protocols (Harland 1991).

### Histological analysis of the musculature

For vibratome sectioning *Xenopus* embryos were sorted according to their fluorescence and fixed in MEMFA overnight. Embryos were then transferred to 25% glutaraldehyde (Roth) and embedded in a 1/10 mixture of gelatin/albumin (4.4% gelatin, 27% bovine serum albumin (BSA), 18% saccharose in 1 × PBS) and 25% glutaraldehyde. Embedded embryos were then trimmed to square blocks and sectioned with a thickness of 40 µm using the Leica Vt1000S vibratom. During sectioning and following immunostaining, sections were kept on coated microscope slides (Roth) in a humid atmosphere.

For immunostaining, the probes were permeabilized in PBS containing 0.2% TritonX for 10 min and subsequently blocked in blocking buffer (1 × PBS + 1% BSA) for one hour at room temperature. The first antibody was diluted in blocking solution and applied to the slides over night at 4 °C. The following primary antibodies were used: anti-GFP (rabbit, Abcam, ab290, 1:1000), anti-Caveolin1 (mouse, BD-Transduction Laboratories, 610406, 1:100), anti-N-CAM (mouse, DSHB, 4d, 1:50). The antibodies were removed by washing three times in 1 × PBS. Subsequently the probes were incubated with secondary antibodies diluted in blocking solution for 2 h at room temperature or overnight at 4 °C. The following antibodies were used: anti-mouse Alexa 594 (Life Technologies, A21203, 1:400), anti-rabbit Alexa 488 (A-21206, 1:400), Phalloidin-TRITC (Sigma-Aldrich (Merck), SLBG6854V, 1:250). Antibodies were removed by washing three times with 1 × PBS and probes were mounted with fluorescence mounting medium (Dako, Deutschland GmbH, Hamburg, Germany). Immunostained sections were imaged with the Leica TCS SP5 microscope (40 × oil objective).

For electron microcopy embryos were fixed in 6.25% glutaraldehyde in 0.1 M cacodylate buffer (0.2 M Cacodylat, pH 7.2 adjust with 0.2 N HCl) overnight. Embryos were washed three times in Cacodylate buffer for 30 min each. For contrasting, samples were incubated in 1% OsO4 in 0.1 M Cocadylate (pH 7.2) for 60–90 min while gently shaking. Osmium was washed out through several washes and overnight incubation in 0.1 M Cocadylate. After contrasting the embryos were embedded in 2.5% Agar–Agar, and then trimmed to square blocks to allow orientation of the embryos during embedding in Spurr’s resin. Prior to embedding in Spurr, embryos were dehydrated in an ascending alcohol-1.4-dioxid series. Dehydration occurred for 30 min each in a EtOH series (50%, 70%) and subsequently in Dioxan for 45 min. Subsequently, the embryos were first infiltrated in a mixture of Spurr’s resin and Dioxan (1:1; 2:1) for 90 min each and then in pure Spurr overnight. The Spurr’s resin was replaced with fresh Spurr and incubated 5–8 h under constant stirring. Finally, embryos were placed in embedding molds and embedded in Spurr for 16 h at 70 °C.

For electron microscopy 50–80 nm ultra-thin sections were prepared using an Ultracut microtome (Reichert) and contrasted with lead acetate and uranyl acetate on Copper-Rhodium (CU/Rh) grids (75 × 300 stiches). For light microscopy 2 µm semi-thin sections were prepared in a LKB-Pyramitom and contrasted in a methylene blue solution at 70 °C for 2–3 min.

### Neural tube explants

Neural tubes were explanted from stage 19–22 embryos as described in^92^. Dissected neural tube explants were cultured in DFA medium (53 mM NaCl, 5 mM Na_2_CO_3_, 4.5 mM Potassium Gluconate, 32 mM Sodium Gluconate, 1 mM CaCl_2_, 1 mM MgSO_4_, 0.5 g/ml BSA, pH 8.3 with 1 M Bicine) on Poly-l-lysine (150 µg/ml, P-1399 Sigma-Aldrich) and Laminin (10 µg/ml, L2020 Sigma-Aldrich) coated chamber slides (Sarstedt) for 12–24 h at 18 °C. Explants were imaged using the Zeiss Axio Observer Z1 inverted microscope (63 × oil objective). Outgrowth as well as morphology of spinal neurons was counted for each explant individually. Axon length, filopodia as well as lamellipodia area was determined using ImageJ. The area covered by lamellipodia was calculated by subtracting the area of the axon (Aa) from the total area including lamelipodia (Ta). Lamellipodia area was then normalized to the total area (Ta) per axon. Number of filopodia per axon was calculated by normalizing the number of counted filopodia (Fn) by the axon length (AL). The growth cone was excluded in both calculations. Filopodia number: (Fn/ AL)*100 µm; Lamellipodia area: (Ta–Aa)/Ta.

### Statistical analysis

All experiments, if not indicated otherwise, were conducted at least three times. The total number of analyzed embryos (n) is indicated for each experiment. Normality of datasets was tested using D’Agostine & Pearson test, Shapiro–Wilk test and Kolmogorov–Smirnov test. Significance was calculated by using either a two-tailed unpaired Student’s t-test and ordinary one-way ANOVA (Dunnett’s multiple comparisons) or Mann–Whitney test (Box plots) (*p-value ≤ 0.05; **p-value ≤ 0.01; ***p-value ≤ 0.001) using Microsoft excel (2013) or GraphPad Prism8. Standard errors of the mean (s.e.m) are shown for each graph, except for supplementary figure SFig.6 where standard deviation (s.d.) is shown. Box plots are presented as Tukey box plots.

## Supplementary information

Supplementary file1

Supplementary file2

Supplementary file3

Supplementary file4

Supplementary file5

Supplementary file6
