# Supplementary material for: Caveolin 1 is required for axonal outgrowth of motor neurons and affects Xenopus neuromuscular development
Source: Sci Rep. 2020 Oct 5;10:16446. doi: 10.1038/s41598-020-73429-x (PMC7536398; doi:10.1038/s41598-020-73429-x)

Caveolin 1 is required for axonal outgrowth of motor neurons and affects *Xenopus* neuromuscular development

Marlen Breuer[1,2], Hanna Berger[1] and Annette Borchers[1,2]

[1] Department of Biology, Molecular Embryology, Philipps-University Marburg, Marburg, Germany

[2] DFG Research Training Group, Membrane Plasticity in Tissue Development and Remodeling, GRK 2213, Philipps-Universität Marburg, Marburg, Germany

*** Corresponding Author:**

Annette Borchers

Department of Biology, Molecular Embryology,

Philipps-University Marburg, Marburg, Germany

E-mail: borchers@uni-marburg.de

Phone: ++49 6421 2826587

Fax: ++49 6421 2821538

Supplementary Figure 1

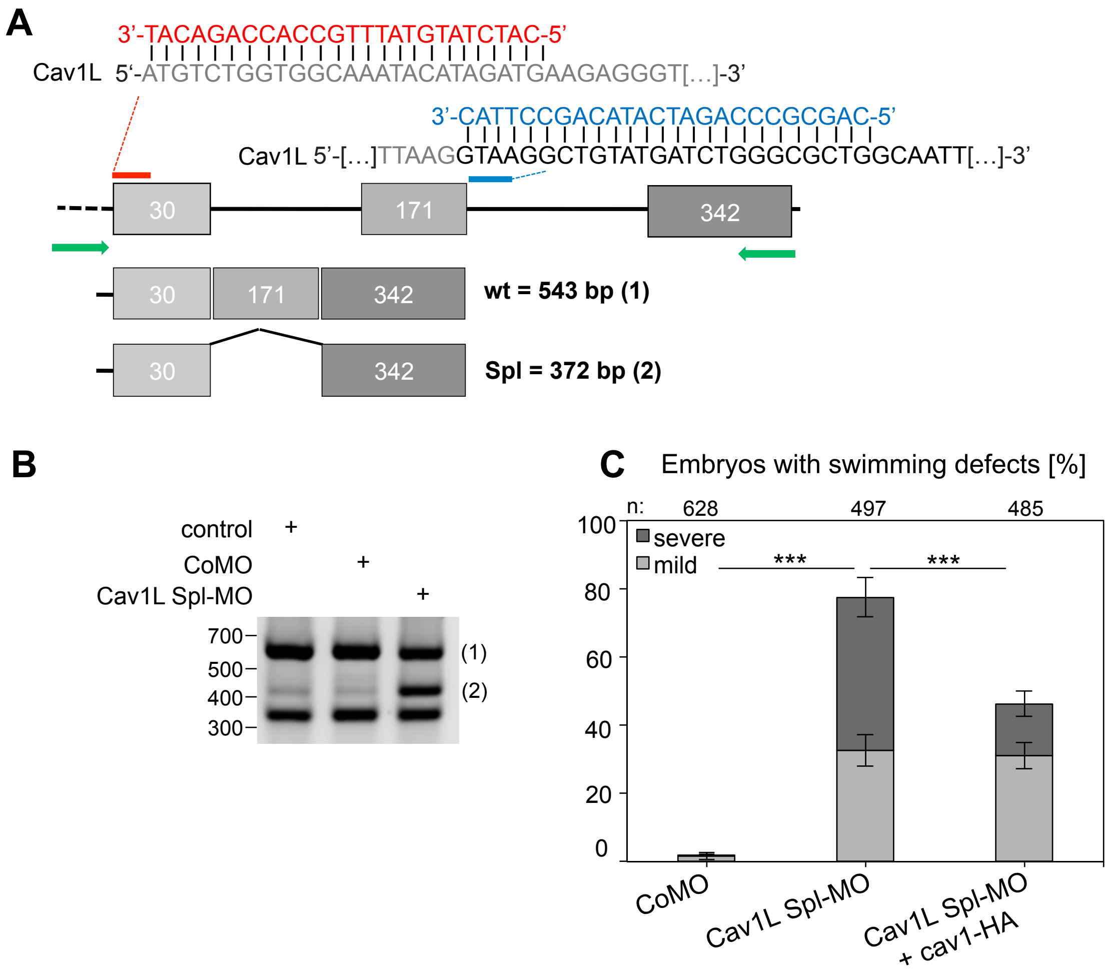

Supplementary Figure 1: Cav1L Morpholino design and functional confirmation. A Schematic of the *cav1L* gene organization and Morpholino design. *Xenopus cav1L* consists of 3 coding exons. For efficient knockdown of Cav1L, two Morpholinos were designed: The translation blocking Morpholino (Cav1L MO, red) binds specifically to the 5' end ATG region of the endogenous *cav1L* RNA (grey). The splice blocking Morpholino (Cav1L Spl-MO, blue) binds the non-coding region of the second intron (black), directly at the exon 2/intron 2 boundary. Different splicing outcomes are shown: (1) A correctly spliced wild type transcript of approx. 543 bp or (2) a deletion of the exon 2 resulting in a truncated transcript of approx. 372 bp in size. **B** The functionality of the Cav1L Spl-MO was tested by RT-PCR using lysates of stage 20 embryos which were injected with 20 ng of Cav1L Spl-MO or a control Morpholino (Co MO). RT-PCR primers are indicated by green arrows in A. Gel electrophoresis conformed the fragments predicted in A, which were verified by sequencing. **C** Percentage of swimming defects of embryos unilaterally injected at the two-cell stage with MO (10 ng) alone or in combination with *cav1L-HA* RNA (200 pg). Data from at least three experiments are presented as the mean ± s.e.m. **p-value ≤ 0.01; ***p-value ≤ 0.001 (Student's t-test comparing the total number of swimming defects).

Supplementary Figure 2

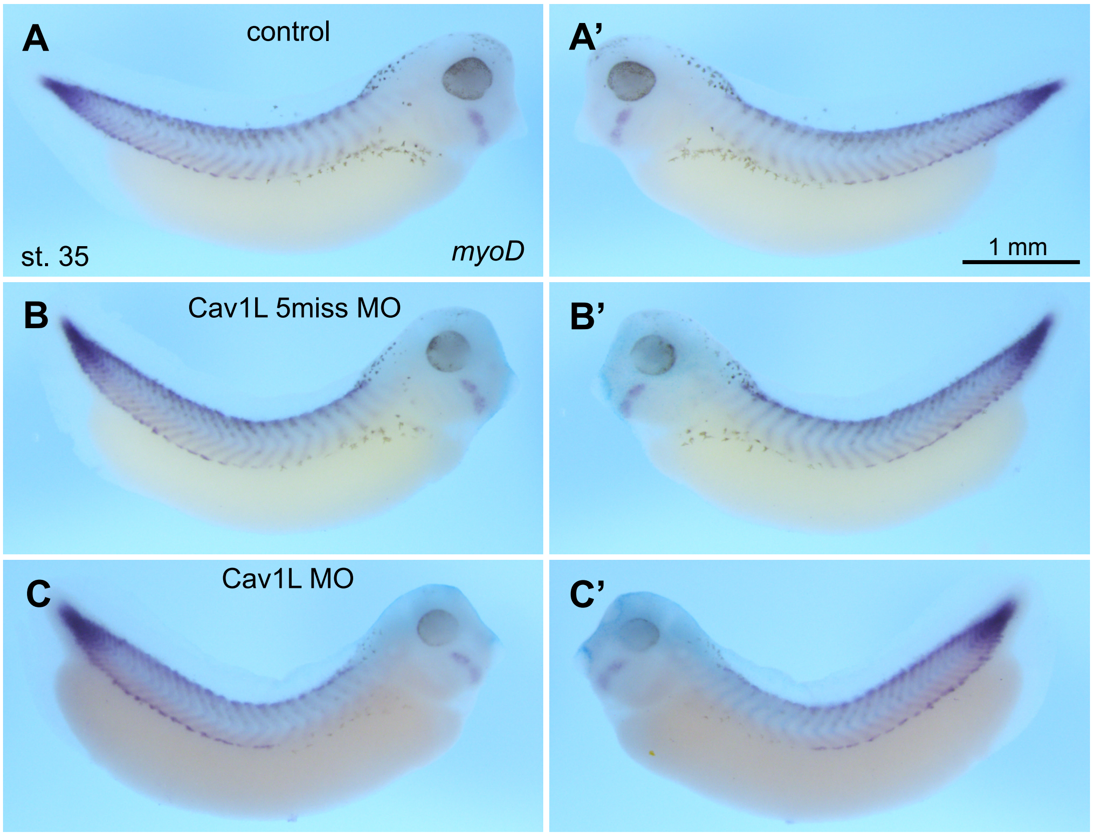

Supplementary Figure 2: *MyoD* expression pattern is not affected by Cav1L Morpholino injection. *MyoD* expression profile in wild type control embryos (A) and embryos injected unilaterally with 10 ng Cav1L-5miss MO (B) or Cav1L MO (C). *LacZ* RNA was co-injected as lineage tracer. Un-injected (A-B) and injected (A’-C’) side is shown. No differences in the *myoD* expression pattern could be observed in embryos injected with Cav1L MO.

Supplementary Figure 3

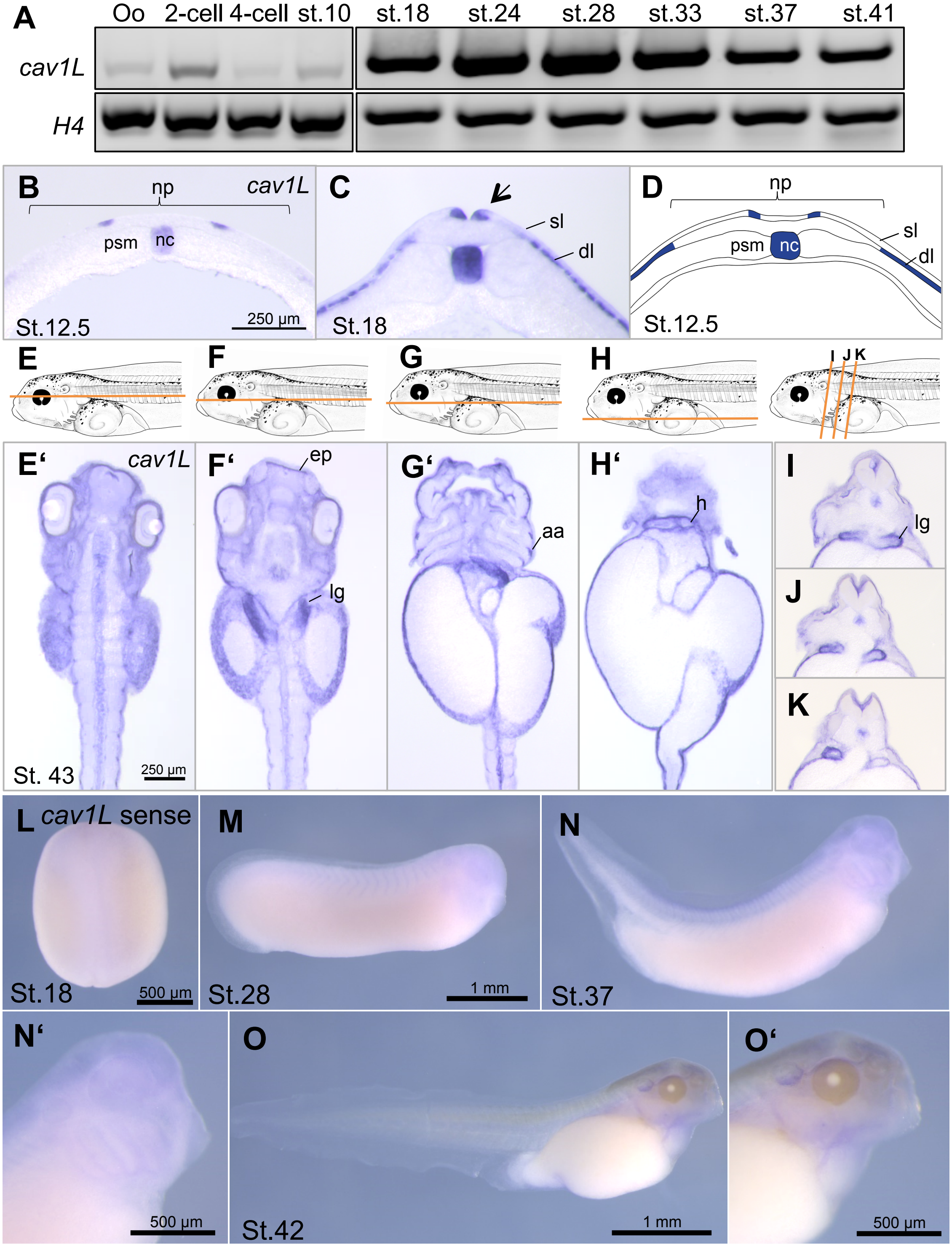

Supplementary Figure 3: *Cav1L* is expressed in the notochord and the nervous system during *Xenopus* development. A Temporal *cav1L* expression was analyzed by reverse transcriptase PCR at different developmental stages. *cav1L* is maternally expressed in the oocyte, zygotic expression starts around stage 10, reaches its peak at stage 28, and declines around stage 33. H4 was used as a loading control. **B-C** Transverse sections of stage 12- 28 embryos shown in Fig. 2 A,C. *cav1L* expression is located in the notochord (nc) in two distinct domains of the sensorial layer (sl) of the neural plate (np) as well as the deep layer (dl) of the epidermis. **D** Schematic view of the expression profile of *cav1L* (violet) in a stage 12.5 embryo. **E-K** Longitudinal (E-H) and transverse (I-K) sections of a stage 43 embryo stained for *cav1L* expression. **E-K** Schematic representation of the cutting position (red line). **E'-K'** *cav1L* is expressed in the epidermis (ep), lung (lg), aortic arches (aa) and the heart (h). Abbreviations: e = eye, nc = notochord, nt = neural tube, so = somite, cg = cement gland. **L-O'** *cav1L* sense probe *in situ* hybridization **L** Embryo at stage 18. **M** Embryo at stage 28. **N** Embryo at stage 37. **N'** Magnification of the embryo shown in N. **O** Embryo at stage 42. **O'** Magnification of the embryo shown in O.

Supplementary Figure 4

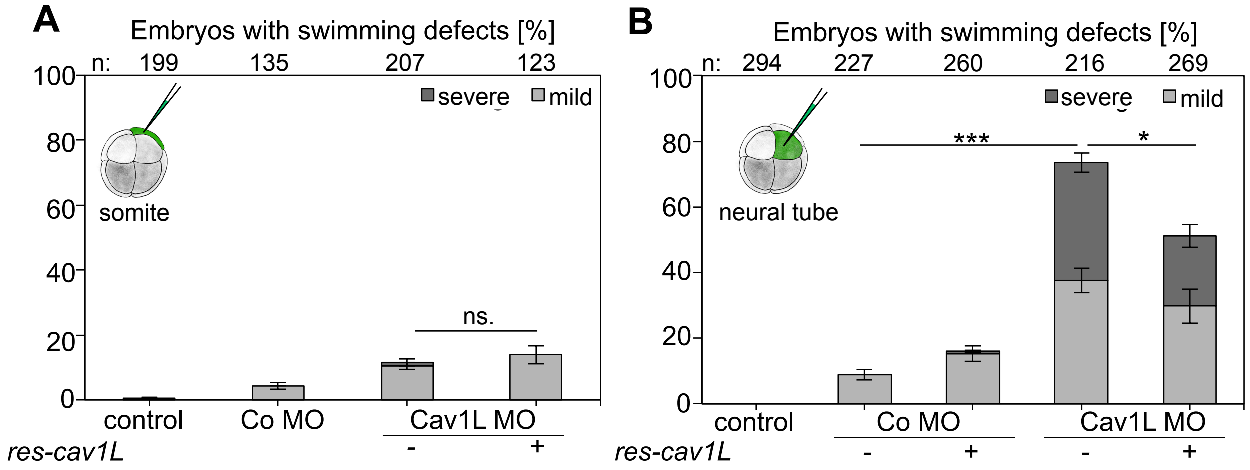

Supplementary Figure 4: Targeted injection of *Cav1L* MO and rescue experiments. 10 ng Cav1L MO alone or in combination with 100 pg *res-cav1L* RNA or *cav1L-HA* RNA were targeted to the somites (A) or the neural tube (B) at the 8-cell stage and the percentage of swimming defects was analyzed at stage 38. Data from at least three experiments are presented as the mean ± s.e.m. * p-value ≤ 0.05; **p-value ≤ 0.01; ***p-value ≤ 0.001 (one-way ANOVA comparing the total number of swimming defects).

Supplementary Figure 5

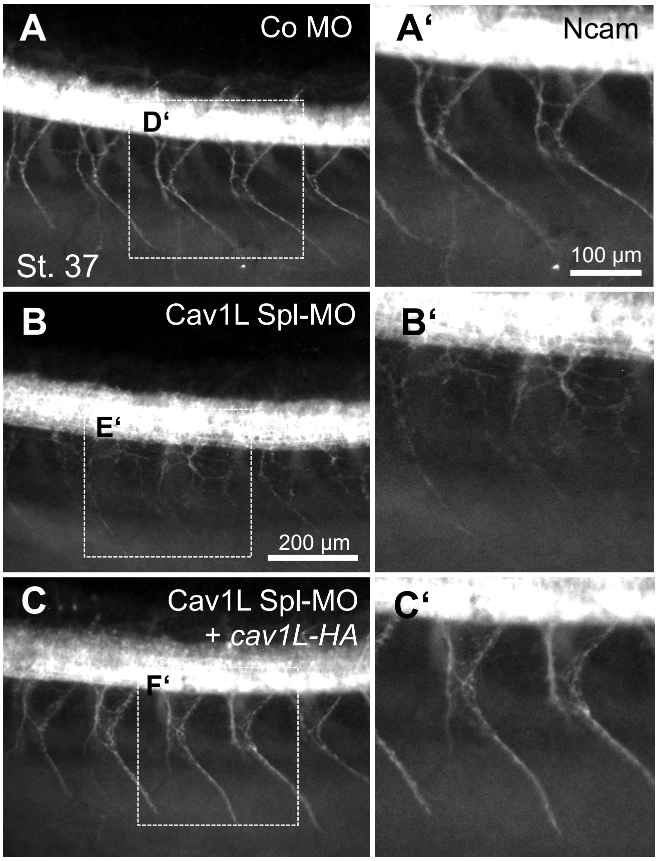

Supplementary Figure 5: Cav1L Spl-MO injection affects motor neuron morphology. A-C Motor neuron morphology of embryos unilaterally injected with 10 ng Co MO, Cav1L MO or Cav1L Spl-MO alone or in combination with 200 pg *cav1L-HA* into two-cell stage embryos. The nervous system of stage 37 embryos was stained by whole mount immunostaining using the neuronal surface marker Ncam; injected side is shown (A-C). **A** Motor neurons of an embryo injected with Co MO. **B** Motor neuron outgrowth is severely affected by Cav1L loss-of-function. **C** Co-expression of *cav1L-HA* RNA rescues motor neuron defects. **A’-C’** Magnified area, highlighted by a dashed box in A-C, is shown.

Supplementary Figure 6

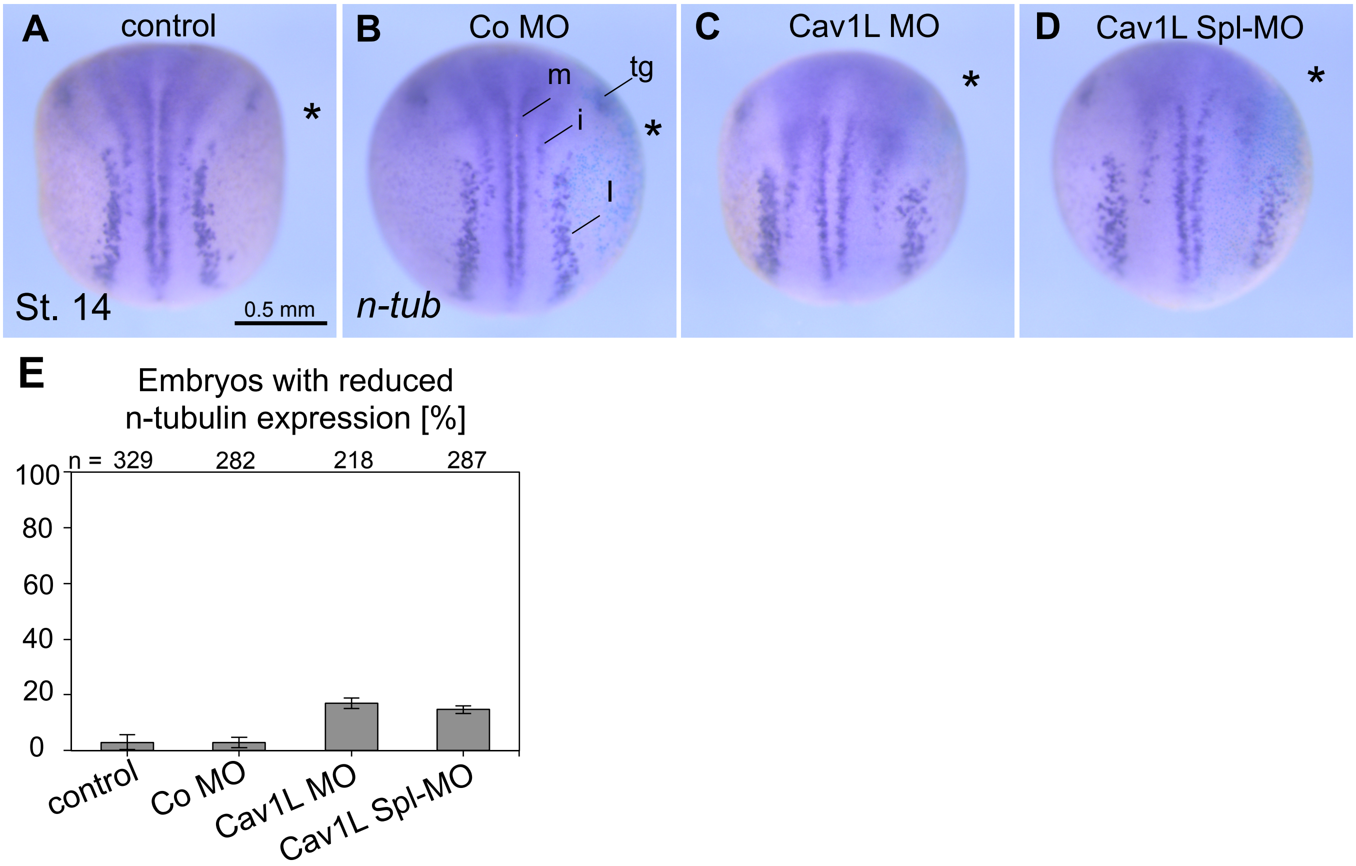

Supplementary Figure 6: Cav1L loss-of-function does not impair *n-tubulin* expression. Expression analysis of *n-tubulin* of embryos unilaterally injected with either 20 ng Co MO, Cav1L MO or Cav1L Spl-MO at the two-cell stage. LacZ staining is shown in blue. M = medial domain, I = intermediate domain, I = lateral domain, tg = trigeminal ganglion. **E** Percentage of embryos with a reduced *n-tubulin* expression is shown from two independent experiments, ± s.d. is shown for each column.

Supplementary Figure 7

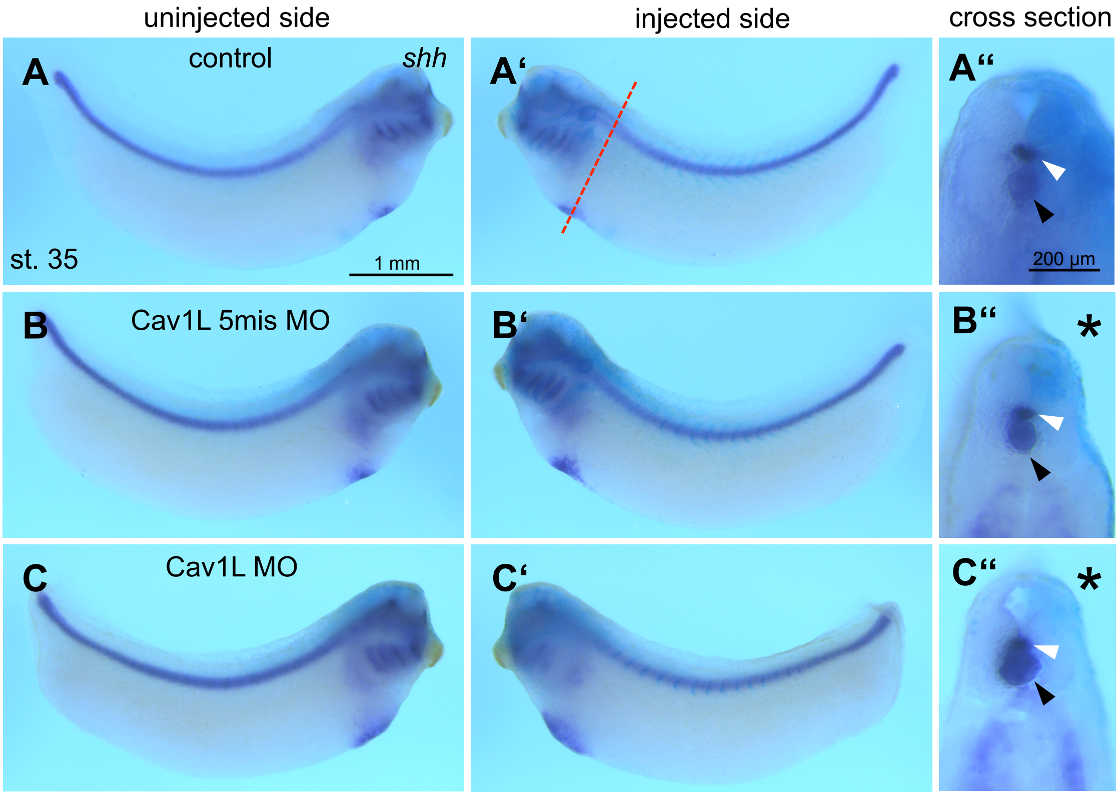

Supplementary Figure 7: Knockdown of Cav1L does not affect *shh* expression in the floorplate. A-C Embryos were injected with 20 ng Cav1L 5miss MO or Cav1L MO and analyzed by *shh* whole mount *in situ* hybridization. Un-injected (A-C) and injected (A'-C') sides are shown. A''-C'' show transverse sections, red dashed lines in A'-C' indicate the section plane. Neither the expression in the floorplate (white arrowhead) nor the notochord (black arrowhead) were affected by Cav1L loss-of-function (asterisks mark the injected side).

Movie 1: Normal swimming behavior of embryos injected with 20 ng Co MO into one blastomere at the two-cell stage.

Movie 2: Embryos injected unilaterally with 20 ng Cav1L MO at the two-cell stage show circular swimming behavior due to the paralysis of the injected site.

Movie 3: Circular swimming movements of embryos unilaterally injected with 20 ng Cav1L Spl-MO at the two-cell stage.

Movie 4: Normal swimming behavior of embryos injected with 10 ng Co MO in both blastomeres of two-cell stage embryos.

Movie 5: Embryos injected with 10 ng Cav1L MO into both blastomeres of two-cell stage embryos. Embryos are completely paralyzed.

Full-length blot presented in **Fig. 1A**

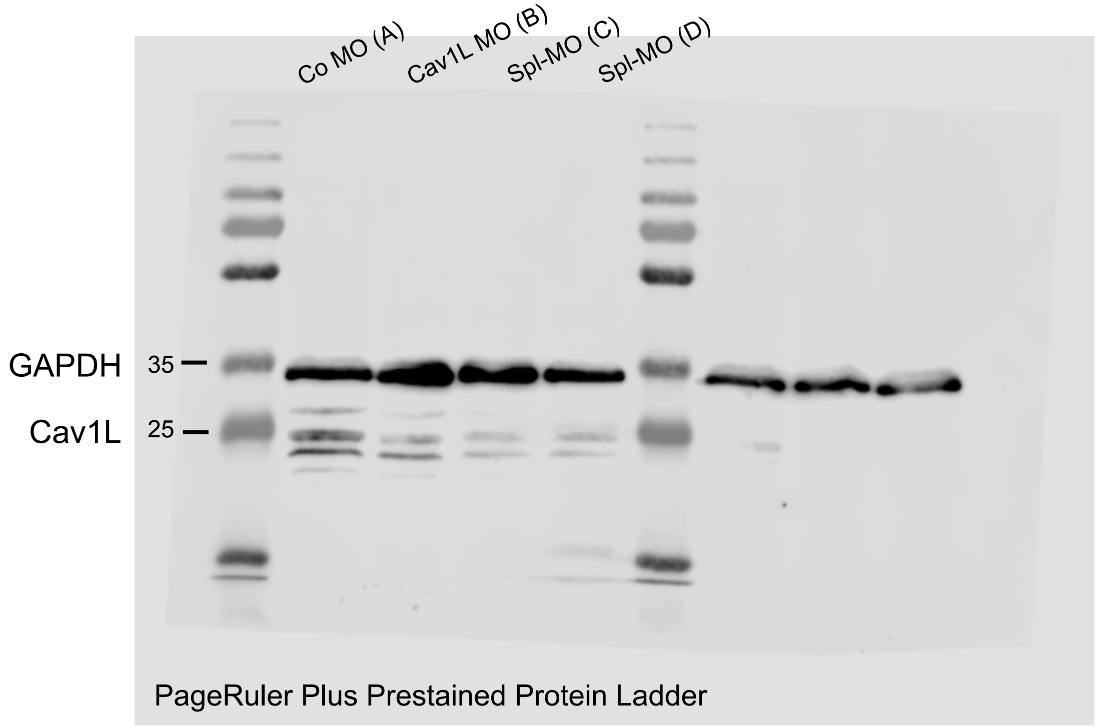

Full-length western blot shown in Figure 1B. **A** Co MO. **B** Cav1L MO. **C** Cav1L Spl-MO shown in Fig.1B. **D** Cav1L Spl-MO not shown and not used for evaluation.

Full-length blot gel presented in **Suppl. Fig. 1B**

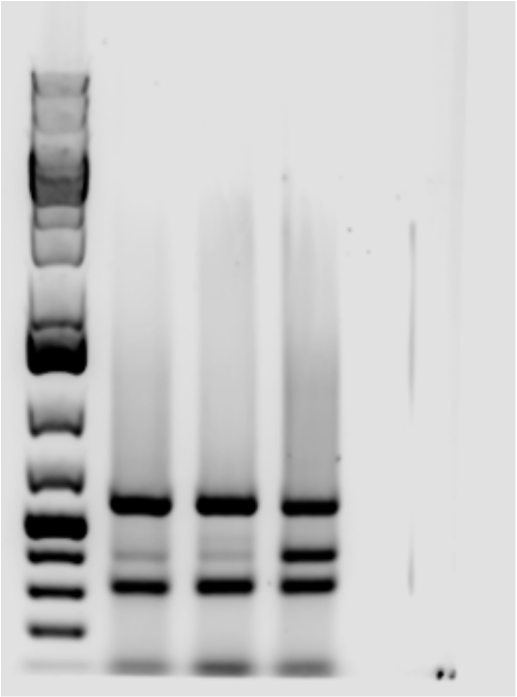

GeneRuler 1 kb DNA ladder

Full-length blot gel presented in **Suppl. Fig. 3**

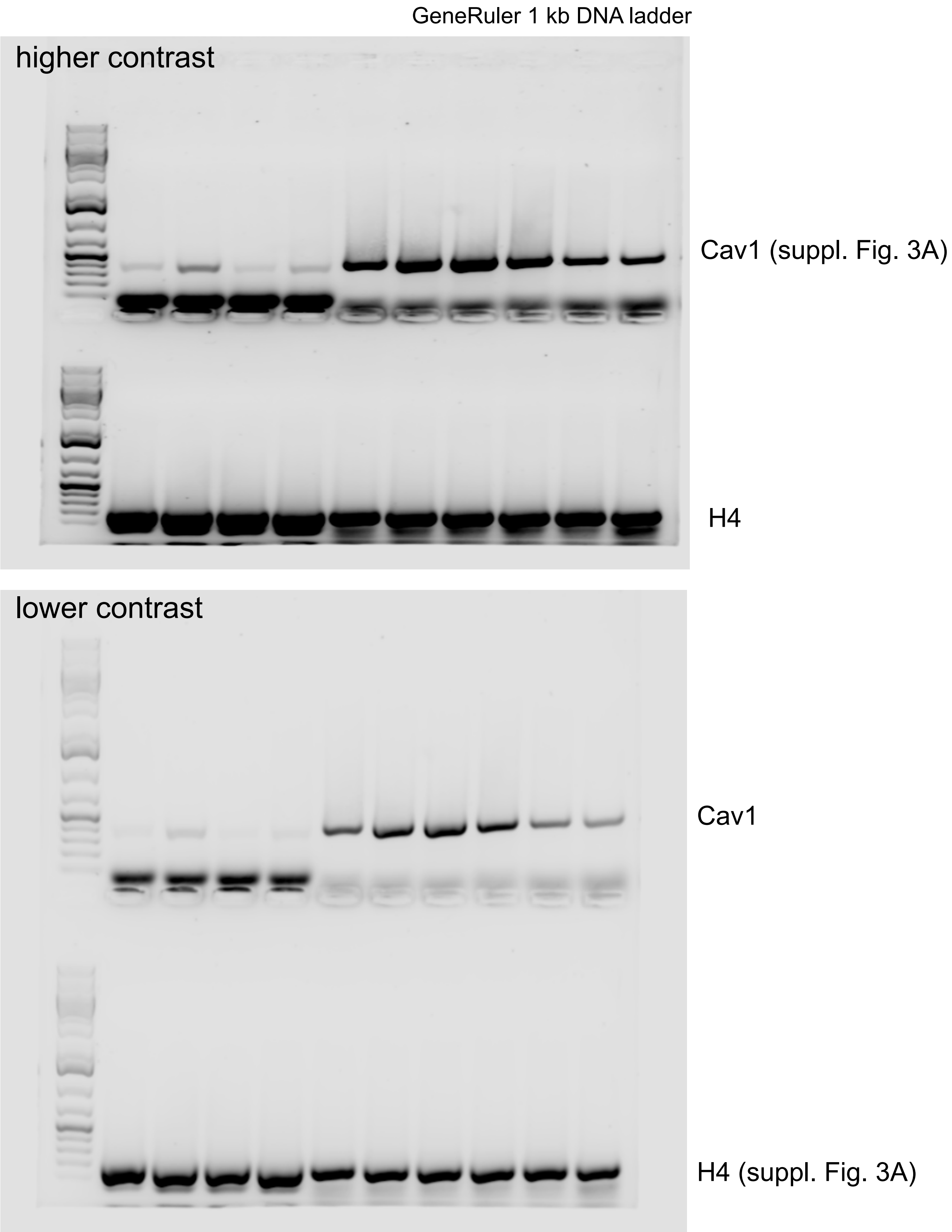

Supplement: Supplementary file 6 — Supplementary file6 [file 41598_2020_73429_MOESM6_ESM.pdf]
